# Network of muscle fibers activation facilitates inter-muscular coordination, adapts to fatigue and reflects muscle function

**DOI:** 10.1038/s42003-023-05204-3

**Published:** 2023-08-30

**Authors:** Sergi Garcia-Retortillo, Carlos Romero-Gómez, Plamen Ch. Ivanov

**Affiliations:** 1 Keck Laboratory for Network Physiology, Department of Physics, Boston University, Boston, MA 02215 USA; 2 Department of Health and Exercise Science, Wake Forest University, Winston-Salem, NC 27190 USA; 3 Complex Systems in Sport, INEFC University of Barcelona, 08038 Barcelona, Spain; 4 Harvard Medical School and Division of Sleep Medicine, Brigham and Women’s Hospital, Boston, MA 02115 USA; 5 Institute of Biophysics and Biomedical Engineering, Bulgarian Academy of Sciences, Acad. Georgi Bonchev Str. Block 21, Sofia, 1113 Bulgaria

**Keywords:** Neurophysiology, Biomarkers

## Abstract

Fundamental movement patterns require continuous skeletal muscle coordination, where muscle fibers with different timing of activation synchronize their dynamics across muscles with distinct functions. It is unknown how muscle fibers integrate as a network to generate and fine tune movements. We investigate how distinct muscle fiber types synchronize across arm and chest muscles, and respond to fatigue during maximal push-up exercise. We uncover that a complex inter-muscular network of muscle fiber cross-frequency interactions underlies push-up movements. The network exhibits hierarchical organization (sub-networks/modules) with specific links strength stratification profile, reflecting distinct functions of muscles involved in push-up movements. We find network reorganization with fatigue where network modules follow distinct phase-space trajectories reflecting their functional role and adaptation to fatigue. Consistent with earlier observations for squat movements under same protocol, our findings point to general principles of inter-muscular coordination for fundamental movements, and open a new area of research, Network Physiology of Exercise.

## Abstract (web summary)

The authors found a complex intermuscular network of synchronous activation among muscle fibers that facilitates movements and adapts to fatigue. Network structure is universal, independent on the specific movement, and reflects the role of muscles involved.

## Introduction

A fundamental question in muscle physiology is how activation and contraction of muscle fibers within a muscle lead to global muscle function and coordination across muscles, which is essential to facilitate a wide range of movements and behaviors. In the last decades, the utilization of reductionist approaches has led to great advances in understanding muscle physiology at the microscopic scale of cellular and molecular level, where investigations focused on the structure and composition of individual muscles in relation to their specific functions. However, it remains unknown how muscle fibers within and across various muscles synchronize their activity to generate coordinated movements. Specifically, we do not know the functional forms and physiological laws of dynamic coupling and network cross-communication among muscle fibers across different muscles, and how these network interactions adapt to different movements and reorganize with training and fatigue.

Movements require coordination among distinct muscle groups. Inter-muscular coordination is associated with a specific distribution of the level of muscle activation among individual muscles to produce given movement^1^. Muscular control during exercise is not limited to switching muscles on and off but includes continuous fine-tuned coordination among muscles with different function and muscle fiber composition. Precise timing and degree of activation of different types of muscle fibers within muscles are essential for intermuscular coordination muscles^2,3^. Coordination across muscles is reflected by the spectral distribution of the surface electromyogram (sEMG). The spectral properties of motor units within a muscle reflect the average conduction velocity (discharge rate) of motor neurons, while the average conduction velocity of an active motor unit is related to muscle fiber-type proportions and fiber contraction characteristics^4–6^. Thus, studying the spectral components of different frequency bands embedded in sEMG signals can reveal important information on motor unit recruitment and on the contribution of different muscle fiber-types to global muscle activation (sEMG) during movement^6–8^.

Studies in recent years have investigated muscle synergies by focusing on frequency-domain cross-coherence among muscles, leading to important findings about intermuscular coordination and the underlying mechanisms of motor control, establishing solid foundations for the field^9–19^, muscle synergy approaches inherently constrain the dimensionality of the neural control to be less than or equal to the number of recorded muscles, and rely on the assumption that all motor neurons from a motor pool (i.e., ensemble of motor neurons innervating a muscle) rigidly receive the same inputs^20^. In other words, the smallest unit of analysis within the current synergistic model is the entire muscle^9,10^. Alternatively, the central nervous system may not directly control global muscle dynamics, but rather controls functional clusters of distinct motor neurons from different motor pools within and across muscles^21,22^. It is hypothesized that functional clusters may be recruited as functional units to control distinct muscles and generate a variety of movements and coordination among muscles. This may explain why topologically distant muscles with different functions and muscle fiber composition can coordinate their activation and synchronize their dynamics to facilitate movements and behaviors.

Further, synergy and coherence-based measures reflect linear aspects of interactions between the same frequency bands (iso-frequency coupling between muscles), and cannot quantify nonlinear dynamic coupling across frequencies^23^. Thus, relevant information regarding the coupling between distinct types of muscle fibers with different firing rates across muscles is ignored^24^. Here we hypothesize that cross-frequency coupling between different frequency bands and continuous dynamic exchange of information within a network of frequency bands^25–30^ may be the carrier mechanism for integrating local and global processes essential to facilitate flexibility to inputs and to generate a rich phase space of behaviors and physiological states.

To further extend current knowledge on muscle coordination and motor control, and to overcome the limitations of synergetic/coherence approaches, we recently developed a method to assess intermuscular cross-frequency interactions among distinct muscles. This led to the discovery of complex hierarchical organization in intermuscular networks of synchronous muscle fiber activation, with functional clusters of subnetworks and modules. We specifically investigated cross-frequency coordination across two leg and two back muscles during a maximal squat test^24^. We uncovered basic physiological principles of intermuscular coordination, and reported the first empirical evidence that different muscle fiber types dynamically synchronize their activity across muscles following distinct patterns of cross-frequency communication that depend on muscle characteristics and on the role muscles play during the squat movement (i.e., primary, secondary, compensatory). Moreover, we established how the global intermuscular network of muscle fiber interactions reorganizes with the transition from rest to exercise and with an accumulation of fatigue during consecutive squat bouts. We discovered that each pair of muscles in the global intermuscular network forms a distinct sub-network that is characterized by a specific signature of cross-frequency communication among distinct muscle fiber types. Further, each subnetwork comprises multiple modules, where each module exhibits a unique profile of links strength stratification reflecting the degree of synchronization (coupling) among muscles during squat movements.

The squat is one of the fundamental movements of the human organism. However, there are other fundamental movement patterns, such as horizontal and vertical push and pulls, rotations or hinges, that need to be investigated to comprehensively understand the mechanisms of coordination muscles and motor control. Each of these basic patterns encompasses a complex variety of distinct muscles and micro-movements, which together form the basis of human movement function. Therefore, we next ask the question of how distinct muscle fibers dynamically synchronize their activation across muscles and respond to fatigue for different fundamental movement patterns. One possible hypothesis is that distinct intermuscular networks of synchronous muscle fiber activation underlie different movement patterns. Alternatively, different movement patterns may be characterized by an intermuscular network with universal characteristics.

Here we specifically select the push-up exercise, a basic horizontal push movement which requires pushing the center of mass away from the ground^31–33^ to test two alternative hypotheses. The first hypothesis is that the push-up moment will show different network organization with distinct patterns of cross-frequency communication, and differentiated response to exercise-induced fatigue compared to the squat movement^24^. This would indicate that each movement pattern involving a specific group of muscles is represented by unique network of muscle fiber interactions, and thus, an infinite number of distinct networks would be needed to characterize/represent the gigantic phase-space of different human movements. An alternative hypothesis is that the recently uncovered profiles of cross-frequency communication among leg and back muscles during squats^24^ will also characterize the network interactions among chest and arm muscles during push-ups. This means that while different movement patterns involve distinct muscles, the underlying network of intermuscular interactions may be characterized by general principles of hierarchical organization (subnetworks and modules) that do not depend on the diversity of the muscles involved, but rather represent the role of the muscles during the movement (major, secondary or compensatory). Thus, an infinite variety of movements involving various combinations of muscles could be represented by a few classes of muscle fiber interaction networks each with a specific hierarchical organization.

Accordingly, the objective of this work is to investigate how distinct muscle fiber types dynamically synchronize with each other and integrate as a network across several chest and arm muscles during a maximal push-up test, and to establish how the network of intermuscular interactions reorganizes with accumulation of fatigue for consecutive push-up bouts. We uncover that the intermuscular network exhibits a complex hierarchical organization of distinct subnetworks and network modules during the push-up movement. Our findings indicate that the network organization dynamically reorganizes in response to fatigue, where distinct subnetworks and modules corresponding to different muscle pairs show a differentiated response of decrease or increase in the degree of coupling among muscle fibers. These empirical results reinforce the findings previously reported for maximal squat tests^24^, and indicate that intermuscular coordination does not depend on the type of fundamental movement pattern, but on the role muscles play during the movement. The Network Physiology framework^34–37^ we employ here opens the perspective for an entire new class of biomarkers based on intermuscular network interactions during distinct fundamental movement patterns, to characterize the effects of training programs, assess levels of fatigue, fitness status and effectiveness of muscle injury rehabilitation.

## Results

We identify and characterize the intermuscular network of muscle fibers interactions during a maximal push-up test, and assess how the network organization reorganizes across repeated exercise sets. We record EMG data from four muscles— left and right pectoralis major (Chest-Left and Chest-Right), and left and right triceps brachii (Arm-Left and Arm-Right).

**Fig. 1:**
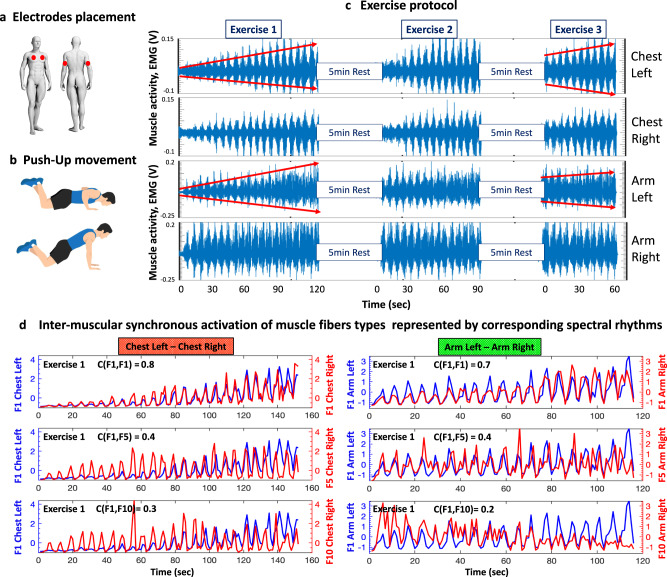
Experimental set up, exercise protocol, raw EMG data and spectral power dynamics of different EMG frequency bands. **a** Red dots mark EMG electrodes placement of selected muscles for simultaneous recording: left and right pectoralis major (ChestL and ChestR); left and right triceps brachii (ArmL and ArmR). **b** Schematic representation of push-ups. This fundamental movement requires continuous coordination of chest and arm muscles activity. **c** Dynamics in EMG signals recorded from each muscle represent the evolution of myoelectrical activity during push-up exercise. The exercise protocol includes three consecutive push-up bouts (Exercise 1, 2 and 3), each performed until exhaustion and separated by 5-min rest periods. EMG amplitude gradually increases with accumulation of fatigue during each exercise bout and higher EMG amplitude at Beginning of Exercise 2 and 3 indicates residual fatigue (“Study design and test protocol” section, Methods). **d** Time series representing spectral power dynamics of EMG frequency bands (corresponding to activation of different muscle fiber types), show synchronous spectral power modulation for different pairs of frequency bands in both muscle pairs ChestL-ChestR and ArmL-ArmR during Exercise 1 for two representative subjects. Blue lines show spectral power of frequency bands Fi from ChestL and ArmL muscles (left vertical axis in the panels), and red lines show spectral power of the bands from ChestR and ArmR muscles (right vertical axis in the panels). During the Beginning and Middle segment of Exercise 1 there is an almost perfect alignment of bursts in the spectral power of muscle fibers from different muscles with practically zero time delay — a behavior observed for frequency bands from both muscle pairs ChestL-ChestR and ArmL-ArmR (thus, our choice to use cross-correlation of the spectral power time series with zero time lag as a measure of coupling, Methods, “Cross-correlations between time series of EMG spectral power in different frequency bands”). Reduction in the degree of synchronous muscle fiber activation during the End segment of Exercise 1 is observed for both muscle pairs and is associated with accumulation of fatigue. This leads to reduction of the cross-correlation measure C(Fi,Fj) that results from two separate effects: (i) time shift of peaks in spectral power related to rhythmicity of the push-ups and (ii) loss of oscillatory activation pattern in the activity of different muscle fibers (frequency bands) and transition to a more stochastic/noisy behavior. Images used in Fig. 1a, b were extracted from the Shutterstock database, and appropriate permissions have been obtained for their usage.

We note that the accumulation of fatigue during an exercise bout is represented by gradual increase in the amplitude of EMG signals from the Arm and Chest muscles as shown in Fig. 1c where the trends in EMG amplitude during Exercise 1 are indicated by red lines. Residual fatigue is evident by the much higher EMG amplitude of Arm and Chest muscles at the start of Exercise 3 compared to the start of Exercise 2 and 1 (Fig. 1c). Additionally, we quantified exercise performance by measuring the number of push-up repetitions. Notably, the number of push-ups significantly decreased for the three consecutive exercise bouts (19.89 ± 6.61, 15.11 ± 6.16, and 13.16 ± 6.36 in Exercise 1, 2, and 3, respectively; Wilcoxon Test comparison of Exc1 vs Exc 2 gives Z = 2.93, *p* = 0.003; Exc1 vs Exc 3, Z = 3.15, *p* = 0.002), reflecting neuromechanical and biochemical alterations (reduced muscle force) induced by Exercise 1.

**Fig. 2:**
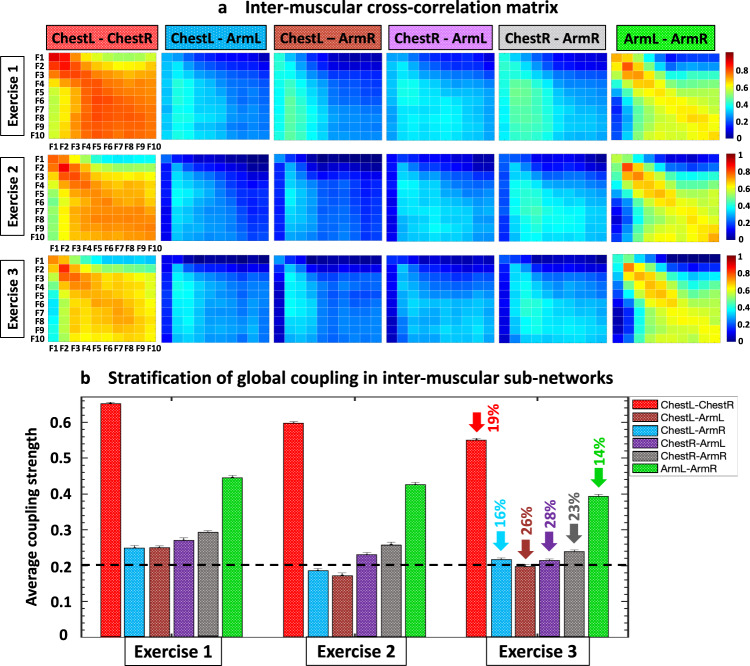
Cross-correlation matrices representing networks of inter-muscular interactions among EMG rhythms during push-up movement. **a** Intermuscular cross-correlation matrices where matrix elements represent the group-average coupling (degree of synchronization, Fig. 1d) between rhythms of myoelectrical activation for all pairs of muscles (ChestL-ChestR, ChestL-ArmL, ChestL-ArmR, ChestR-ArmL, ChestR-ArmR, ArmL-ArmR). Each matrix represents a subnetwork of intermuscular interactions among myoelectrical rhythms (10 frequency bands F1, F2, …, F10 with equal width of 19.5 Hz in the intervals [5-45 Hz] and [55-215 Hz]; for each pair of muscles. The F_i_ frequency bands correspond to the range of activity of different types of muscle fibers in each Chest and Arm muscle, i.e., F1 = [5-24.5 Hz], F2 = [25-44.5 Hz], F3 = [55-74.5 Hz], F4 = [75-94.5 Hz], F5 = [95-114.5 Hz], F6 = [115-134.5 Hz], F7 = [135-154.5 Hz], F8 = [155-174.5 Hz], F9 = [175-194.5 Hz] and F10 = [195-214.5 Hz]. Matrix rows show myoelectrical rhythms derived from the first muscle and matrix columns correspond to the rhythms in the second muscle for each pair; color code in vertical bars indicates coupling strength (based on cross-correlation C(F_i_,F_j_) of the spectral power amplitude of F_i_ and F_j,_ Fig. 1d). Heterogeneity of the matrices indicates complex organization of network links (coupling strength). **b** The average coupling strength for each intermuscular subnetwork exhibits a pronounced stratification pattern during Exercise 1, 2 and 3—bars represent the average value of all matrix elements for each matrix in (**a**), i.e., average links strength for each subnetwork. Error bars on top of each bar indicate the standard error; horizontal black dotted line marks the threshold of physiological significance for network interactions of myoelectrical rhythms across muscles based on a surrogate test where rhythms from different subjects are randomly coupled (“Fourier phase randomization surrogate test and significance threshold for links strength in networks of intermuscular interactions”, Methods).

**Fig. 3:**
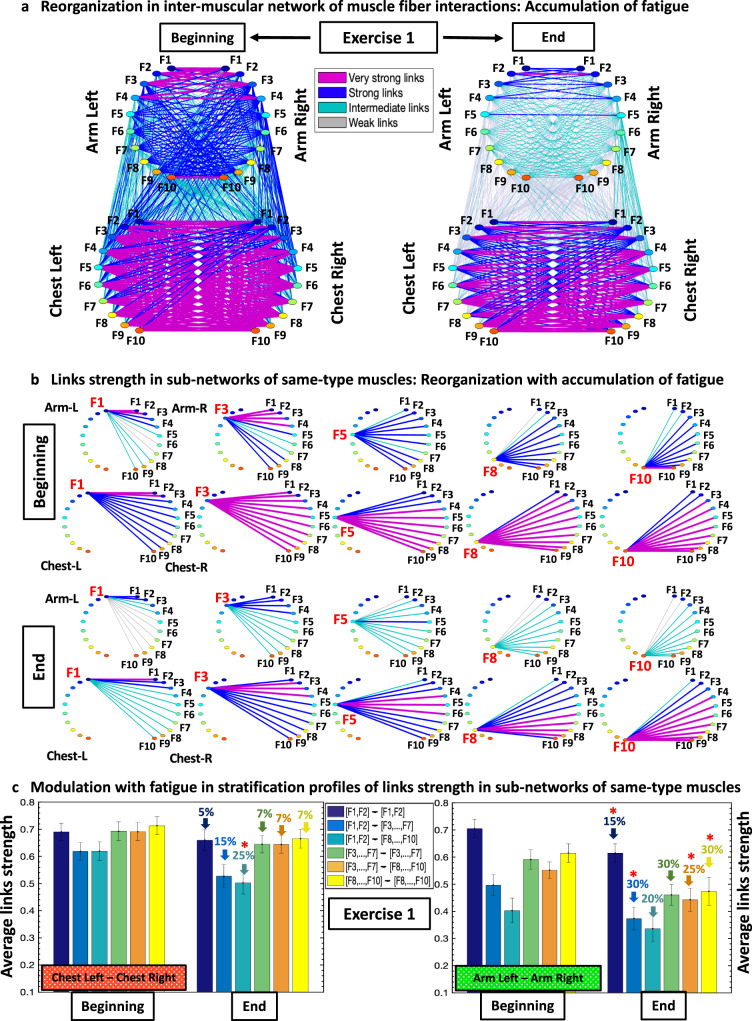
Interaction networks of myoelectrical rhythms across muscles and their evolution with accumulation of fatigue during exercise. **a** Dynamic networks of intermuscular interactions between major muscles involved in push-up movement show network reorganization with accumulation of fatigue during Exercise 1. Group-averaged network maps derived from the cross-correlation matrices for Exercise 1 (Fig. 2a), where network links are the cross-correlation matrix elements and represent the coupling strength (degree of synchronization) between myoelectrical rhythms (frequency bands F_i_) corresponding to the degree of synchronous activity of muscle fibers from different muscles. Networks are derived from data corresponding the Beginning (first third) and End (last third) segments of Exercise 1. Each muscle is shown as semicircle with color nodes for the different frequency bands F_i_ (“Spectral decomposition”, Methods) within the muscle. Line color and width mark the links strength (Section “Intermuscular interaction networks”, Methods). Intermuscular interactions among muscle fibers form a multiplex network with pronounced heterogeneity characterized by distinct topology and hierarchical organization of links strength for subnetworks representing pairs of same- and different-type muscles. **b** Same-type muscle ChestL-ChestR and ArmL-ArmR subnetworks play main role in the push-up movement, exhibit hierarchical structure of links strength, and reorganize with accumulation of fatigue from Beginning to End segments of Exercise 1. Diagrams show distinct modules in these subnetworks for low (F1, F3), intermediate (F5) and high (F8, F10) frequency bands. These modules are obtained from the network maps in (a) using the same color code. **c** Stratification profiles of links strength for the ChestL-ChestR and ArmL-ArmR subnetworks reorganize with accumulation of fatigue from Beginning to End of Exercise 1. Six bars in each profile correspond to the group-average links strength of all muscle fiber interaction modules within each subnetwork. Error bars indicate standard error. Red stars mark statistically significant differences in links strength for all modules comparing Beginning vs End (Wilcoxon test p values < 0.05).

Further, the experimental data we show in Fig. 1d demonstrates the relation between the cross-frequency coupling function and accumulation of fatigue (Fig. 2). In the Beginning segment of Exercise 1 the spectral power amplitude of different EMG frequency bands is low and bursts in spectral power are well synchronized across frequency bands (synchronous movement of the red and blue lines in Fig. 1d). In contrast, the End segment of Exercise 1 exhibits a dramatic increase in the spectral power amplitude of frequency bands (an indicator of fatigue accumulation related to the increase of EMG amplitude with fatigue in Fig. 1c), which is notably paralleled by a remarkable loss of coordination/synchrony of bursts across frequency bands (shift of peaks between the red and blue lines in Fig. 1d). Such loss of synchrony in the activity of different muscle fiber types in the End segment of the exercise bout is quantified by a significant decline in the degree of cross-correlation (Fig. 2), and thus, a corresponding decline of network links strength with fatigue (Fig. 3). Remarkably, such loss of synchrony in bursting activity of muscle fibers and corresponding change in network links strength is consistently observed for the links in all modules of the ChestL-ChestR and ArmL-ArmR sub-networks (left and right panel in Fig. 1d and Fig. 3).

We find that the intermuscular network during the push-up movement is characterized by an ensemble of interaction subnetworks (distinct heterogenous matrices) representing all pairs of muscles, where each subnetwork exhibits a specific pattern of synchronization (hierarchical structure in network links strength) among myoelectrical rhythms associated with different types of muscle fibers. With the accumulation of fatigue for repeated bouts of Exercise 1, 2 and 3, our results uncover a complex hierarchical reorganization in the coupling strength of muscle fibers where distinct subnetworks within the entire intermuscular network respond in a targeted way to overcome reduced motor neuron excitability. These general observations reflect a complex mechanism of intermuscular interactions mediated through synchronous activation of distinct muscle fiber types.

### Intermuscular networks and reorganization with accumulation of fatigue during Exercise 1

To quantify intermuscular networks of muscle fiber interactions, we map the cross-correlation matrices in Fig. 2a into dynamic networks where nodes correspond to distinct myoelectrical rhythms representing the activity of distinct muscle fibers, and links represent the coupling strength among rhythms (frequency bands F_i_) across muscles (Fig. 3a).

To identify the effects of accumulation of fatigue during Exercise 1, we split the exercise bout into Beginning segment (first third) and End segment (last third), and we apply our method to quantify accumulation of fatigue in intermuscular cross-frequency interactions among distinct muscle fiber types (different EMG frequency bands) (Fig. 3). Remarkably, we find that with accumulation of fatigue within an exercise bout there is a decline in both ChestL-ChestR and ArmL-ArmR subnetwork interactions, which is more pronounced for the ArmL-ArmR subnetwork (Fig. 3a). At the Beginning segment of Exercise 1, ChestL-ChestR and ArmL-ArmR subnetwork modules representing the interactions of low-low ([F1-F2]—[F1-F2]), intermediate-intermediate ([F3-F7]-[F3-F7]), intermediate-high ([F3-F7]-[F8-10]) and high-high ([F8-F10]—[F8-F10]) frequency bands exhibit dominant links strength, while modules corresponding to low-intermediate ([F1-F2]—[F3-F7]) and low-high ([F1-F2]—[F8-F10]) frequency bands show weaker links strength (Fig. 3b, c). The unique interaction profile in Fig. 3c indicates that slow type-I muscle fibers in the ChestL or ArmL muscle predominantly coordinate (synchronize activation) with slow type-I fibers in the ChestR or ArmR muscle, while intermediate type IIa fibers coordinate with both type IIa and fast type IIb fibers. In contrast, slow type-I muscle fibers exhibit a lower degree of coordination (network links strength) with fast type-II fibers within the ChestL-ChestR and ArmL-ArmR subnetworks (Fig. 3b, c). Note that at the Beginning segment the ArmL-ArmR subnetwork shows lower coordination (weaker links) and higher stratification of links strength compared to the ChestL-ChestR subnetwork.

With the accumulation of fatigue at the End segment of Exercise 1, the shape of the ChestL-ChestR links strength profile is preserved, however, with (i) reduced coupling strength between all types of muscle fibers and (ii) increased profile stratification due to more pronounced decline in interactions between slow type-I and fast type-II muscle fibers (third bar of the profile in Fig. 3c). Similarly, the general shape of the ArmL-ArmR links strength profile is preserved with an accumulation of fatigue during Exercise 1 (comparing bar charts for Beginning vs End segment in Fig. 3c), however, with no change in the stratification of the profile (preserved profile spread) due to homogeneous decline in the coupling strength among all types of muscle fibers with fatigue. These observations for the different subnetwork modules are further confirmed with a more detailed analysis of the strength of individual links in the ChestL-ChestR and ArmL-ArmR subnetworks.

**Fig. 4:**
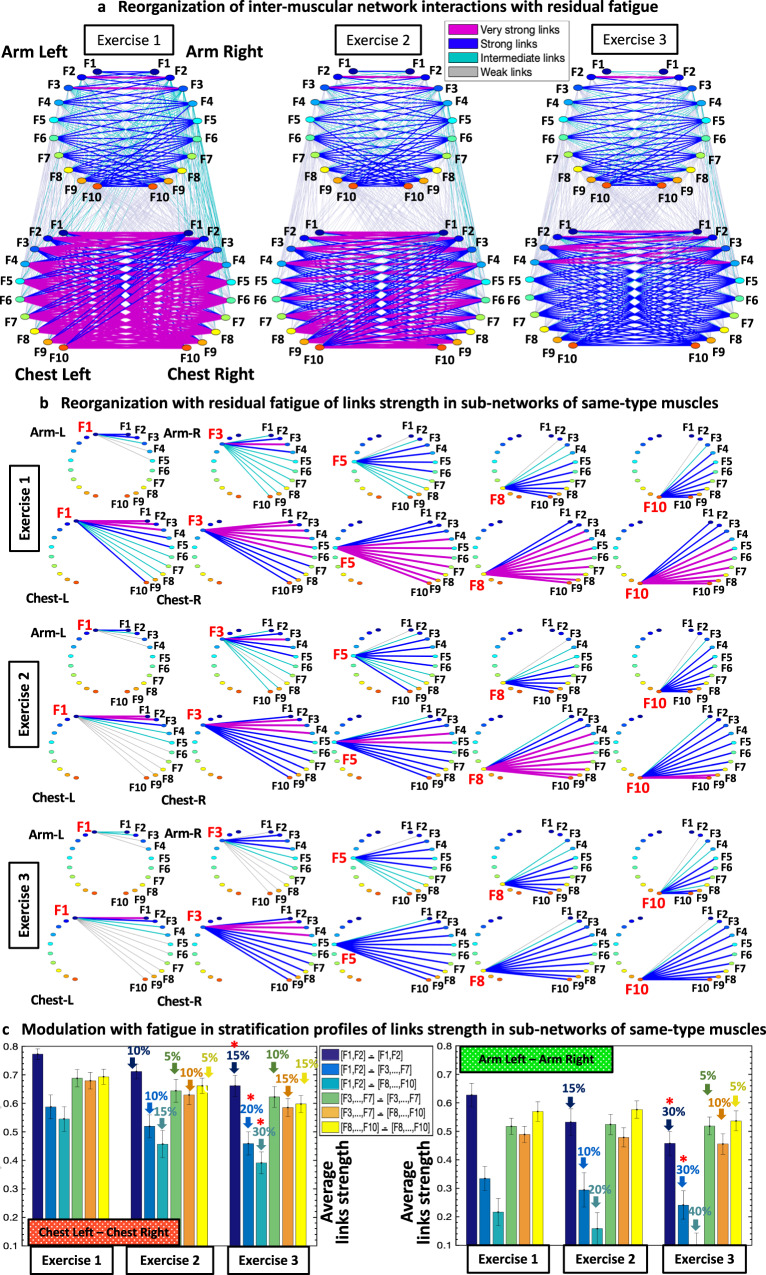
Interactions networks of myoelectrical rhythms across muscles and their evolution with fatigue. **a** Dynamic networks of intermuscular interactions between major muscles involved in push-up movement show network reorganization with progression of fatigue for repeated bouts of exercise. Group-averaged network maps derived from the cross-correlation matrices for Exercise 1, 2 and 3 shown in Fig. 2a, where network links are the cross-correlation matrix elements and represent the coupling strength (degree of synchronization) between myoelectrical rhythms (frequency bands) corresponding to the degree of synchronous activity of muscle fibers from different muscles. Intermuscular interactions form a multiplex network with pronounced heterogeneity characterized by distinct topology and hierarchical organization of links strength for subnetworks representing pairs of same- and different-type muscles. Each muscle is shown as semicircle with color nodes for the different frequency bands (“Cross-correlations between time series of EMG spectral power in different frequency bands”, Methods) within the muscle. Line color and width mark the links strength (“Intermuscular interaction networks”, Methods). **b** Same-type muscle ChestL-ChestR and ArmL-ArmR subnetworks play main role in the push-up movement, exhibit hierarchical structure of links strength, and reorganize with fatigue during Exercise 2 and 3. Diagrams show modules in these subnetworks for low (F1, F3), intermediate (F5) and high (F8, F10) frequency bands. These modules are obtained from the network maps in (**a**) using the same color code. **c** Stratification profiles of links strength for the ChestL-ChestR and ArmL-ArmR subnetworks. Stratification profiles reorganize in response to fatigue during consecutive exercise bouts. Six bars in each profile for the ChestL-ChestR and ArmL-ArmR subnetwork correspond to the group-average links strength of all muscle fiber interaction modules within each subnetwork. Error bars indicate standard error. Red stars mark statistically significant differences in links strength for all modules comparing Exercise 3 vs Exercise 1 (Wilcoxon test *p* values with multiple tests Bonferroni correction < 0.025).

Next, we repeat our analysis for the entire bout of Exercise 1. We find that the same-type muscle ChestL-ChestR and ArmL-ArmR subnetworks exhibit strong and intermediate links, in contrast to the subnetworks of different-type Chest-Arm muscles with weaker coupling among myoelectrical rhythms (Fig. 4a). To probe the hierarchical structure of network links strength, we dissect the entire network into separate subnetwork modules for low (F1, F2, F3), intermediate (F4, F5, F6, F7) and high (F8, F9, F10) frequency EMG rhythms. We find that within same-type muscle ChestL-ChestR and ArmL-ArmR subnetworks, all network modules show consistent stratification in links strength—i.e., links are stronger for matching frequency bands and gradually decline for more distant frequency bands from the two muscles. This effect is more pronounced for the ArmL-ArmR subnetwork, where low frequencies (F1-F3) in the ArmL are highly synchronized with low frequencies in ArmR; intermediate frequencies (F5) in ArmL communicate to intermediate and high frequencies in ArmR, and high frequencies (F8-F10) in ArmL communicate to intermediate and high frequencies in BackR (Fig. 4b).

**Fig. 5:**
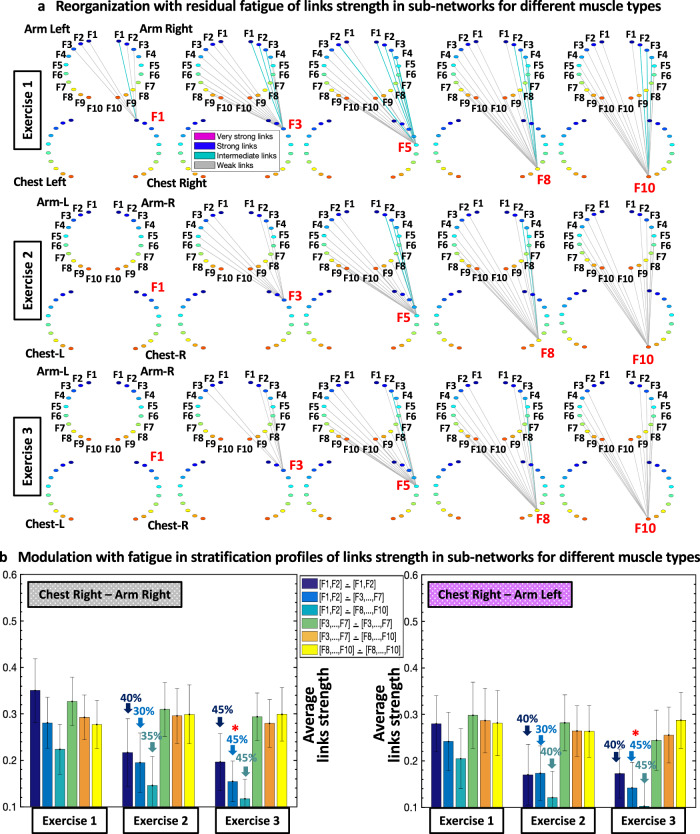
Subnetworks of synchronous activity among myoelectrical rhythms for pairs of different muscle types and their reorganization with fatigue. **a** Muscle fiber interactions for the different-type muscle ChestR-ArmR and ChestR-ArmL subnetworks. These subnetworks play supportive/secondary role in push-up movement and exhibit specific hierarchical organization of links strength. Shown are the subnetwork modules for low (F1, F3), intermediate (F5) and high (F8, F10) frequency bands extracted from the network maps in Fig. 4a using the same color code. The other two subnetworks ChestL-ArmL and ChestL-ArmR of different muscle types exhibit similar network structure (modules) and reorganization with fatigue for repeated exercise bouts (matrices in Fig. 2a). **b** Links strength stratification profiles for the ChestR-ArmR and ChestR-ArmL subnetworks and profile reorganization for repeated exercise bouts with fatigue. Six bars in each profile correspond to the group-average links strength of all muscle fiber interaction modules within each subnetwork. Error bars indicate standard error. Red stars mark statistically significant differences in links strength for all modules comparing Exercise 3 vs. Exercise 1 (Wilcoxon test *p* values with multiple tests Bonferroni correction < 0.025).

As for different-type muscle subnetworks, we find that during Exercise 1 each network module in the ChestR-ArmR and ChestR-ArmL subnetworks is characterized by different average link strength and by a specific topological organization for links of different strength (Fig. 5a)—stronger and more heterogenous links in the F1 module compared to weaker and more homogeneous links in the F10 module, reflecting a distinct pattern of cross-communication and synchronization of muscle fibers in different-type muscle subnetworks compared to the same-type muscle subnetworks (Fig. 4).

Next, to dissect the intermuscular network and quantify the stratification patterns observed for the links strength of the modules embedded in the same-type muscles subnetworks (ChestL-ChestR and ArmL-BackR; Fig. 4c) and in the different-type muscle subnetworks (ChestR-ArmR and ChestR-ArmL; Fig. 5b), we compute the average coupling strength for distinct sub-groups of links within modules. We find that subnetworks of same-type muscle ChestL-ChestR and ArmL-BackR exhibit a stratification profile with decline in average links strength for the modules corresponding to low-low ([F1-F2]—[F1-F2]), low-intermediate ([F1-F2]—[F3,…,F7]) and low-high ([F1-F2]—[F8,…,F10]) frequency bands interactions (first three bars of the stratification profile in Fig. 3c), and stronger links (higher bars) for the subnetwork modules corresponding to intermediate-intermediate ([F3,…,F7]—[F3,…,F7]), intermediate-high ([F3,…,F7]—[F8,…,F10]) and high-high ([F8,…,F10]—[F8,…,F10]) frequency bands interactions (last three bars of the stratification profile in Fig. 4c).

Regarding subnetworks of different muscle type, ChestR-ArmR and ChestR-ArmL exhibit similar interaction profiles during Exercise 1 — i.e., decline in average links strength for the subnetwork modules corresponding to low-low ([F1-F2]—[F1-F2]), low-intermediate ([F1-F2]—[F3,…,F7]) and low-high ([F1-F2]—[F8,…,F10]) frequency bands interactions (first three bars in the profile, Fig. 3b), and the same up-down-down pattern for intermediate-intermediate ([F3,…,F7]—[F3,…,F7]), intermediate-high ([F3,…,F7]—[F8,…,F10]) and high-high ([F8,…,F10]—[F8,…,F10]) frequency bands interactions (last three bars in the profile, Fig. 5b).

### Intermuscular networks of muscle fiber interactions and reorganization with residual fatigue

Next, we study how network organization and the profiles of network links strength within subnetworks for Exercise 1 reorganizes with repeated Exercise 2 and 3 for subnetworks of same-type (Fig. 4b, c) and different-type muscles (Fig. 5a, b). Our findings reveal that the global intermuscular network undergoes complex hierarchical reorganization, where subnetworks representing muscle pairs with distinct role during the push-up movement, exhibit differentiated response with accumulation of fatigue (Fig. 4a).

#### Same-type muscle subnetworks

All modules in the ChestL-ChestR subnetwork remarkably reorganize due to pronounced fatigue effects provoked by Exercise 1. During Exercise 2 and 3, links in the ChestL-ChestR subnetwork modules exhibit (i) decreased coupling strength among frequency bands and (ii) increased stratification in links strength — links are stronger for matching frequency bands and progressively decrease for more distant frequency bands from the two muscles (Fig. 4b). Specifically, the stratification in links strength increases for all modules in the ChestL-ChestR subnetwork during Exercise 2 and 3 — from very strong and strong links during Exercise 1, to the entire spectrum of very strong to intermediate and weak links during Exercise 2 and 3. In contrast, while ArmL-ArmR subnetwork interactions become weaker during Exercise 2 and 3, the link strength stratification for all modules remains similar — i.e., coupling strength is similarly reduced for all ArmL-ArmR subnetwork links (Fig. 4b).

In order to quantify the hierarchical organization of links strength in network modules observed in the same-type muscle ChestL-ChestR and ArmL-ArmR subnetworks (Fig. 4b), we next obtain the link strength stratification profiles for each subnetwork, and we track the evolution of the stratification profiles in response to fatigue for Exercise 2 and Exercise 3 (Fig. 4c). Our empirical observations show that with accumulation of fatigue the average links strength declines for all modules in the ChestL-ChestR subnetwork. Further, this decline in links strength is associated with increased stratification in the interaction profile of the ChestL-ChestR subnetwork, due to a more pronounced reduction for low-high ([F1-F2]—[F8,…,F10]) frequency bands (3^rd^ bar in the profile in Fig. 4c; Exercise 3 vs. Exercise 1 Wilcoxon Test, *p* < 0.03).

In contrast to the ChestL-ChestR subnetwork, links strength in the modules of the ArmL-ArmR subnetwork is reduced during Exercise 2 and 3, however, preserving a similar form of the stratification profile — i.e., coupling strength is similarly reduced for low-low ([F1-F2]—[F1-F2]), low-intermediate ([F1-F2]—[F3,…,F7]) and low-high ([F1-F2]—[F8,…,F10]) frequency bands (first three bars in the profile in Fig. 4c; Exercise 3 vs Exercise 1 Wilcoxon Test, *p* < 0.05). Our observations indicate distinct response to fatigue of same-type muscle ChestL-ChestR and ArmL-ArmR subnetworks, where network reorganization and the associated stratification profile of links strength undergo different trajectories corresponding to the different role these muscles play during push-up movement (primary or secondary).

#### Subnetworks of different muscle types

Since our observation in Fig. 4 demonstrate that the ChestL-ChestR and ArmL-ArmR subnetworks exhibit different characteristics (connectivity and links strength stratification) and response to fatigue that are associated with their specific role (primary vs. secondary) during push-ups movements, we next study muscle fibers interactions between pairs of different-type muscles (Chest-Arm; Fig. 5) and quantify their contribution to the hierarchical structure of the global intermuscular network. With progression of fatigue for Exercise 2 and Exercise 3, the average link strength decreases heterogeneously for the different modules (Fig. 5a), indicating a hierarchical reorganization in response to accumulation of fatigue that is different compared to the response observed for interactions in the same-type muscle subnetworks (Fig. 4b).

To assess the response to fatigue of muscle fiber interactions within the different-type muscle ChestR-ArmR and ChestR-ArmL subnetworks, we obtain stratification profiles representing the average links strength for all network modules embedded in the subnetworks, and we quantify the evolution of these profiles (Fig. 5b). With progression of fatigue for consecutive Exercise 2 and 3, the general shape of the interaction profiles for both ChestR-ArmR and ChestR-ArmL subnetworks is preserved, however, the average links strength corresponding to the subnetwork modules of low-low, low-intermediate and low-high frequency band interactions (first three bars in the profiles, Fig. 5b) significantly decreases (Exercise 3 vs. Exercise 1 Wilcoxon Test, *p* < 0.05). Notably, the different-type muscles subnetworks (Fig. 5b) exhibit distinct links strength stratification profiles and modulation with fatigue compared to the interaction profiles of the same-type muscles subnetworks shown in Fig. 4c — while the average links strength of same-type muscle subnetworks decline with fatigue across all modules (six bars in the profile; Fig. 4c), link strength in the different-type muscle subnetworks only decreases for the first 3 bars in the profile (Fig. 5b). We observe a similar network structure and evolution during Exercise 2 and Exercise 3 for the other two subnetworks ChestL-ArmL and ChestL-ArmR of different muscle types (matrices shown in Fig. 2a).

**Fig. 6:**
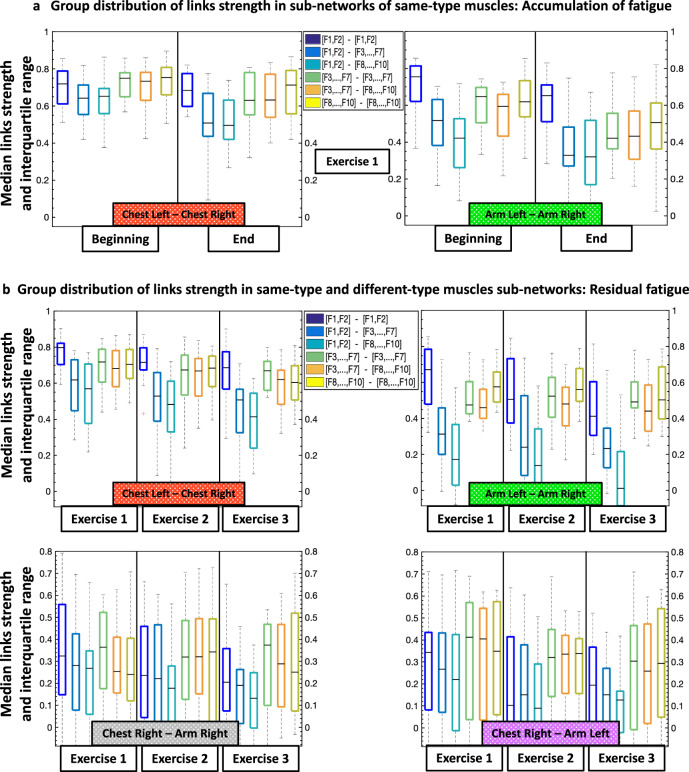
Data statistics for average links strength in subnetwork modules and effects of accumulation and residual fatigue. Boxplots display the median links strength and interquartile range for the average links strength of network modules within subnetworks of different muscle pairs obtained separately for each subject in the database. **a** Boxplots representing subject statistics for the average links strength of the six modules (shown in different box color) in the same-type muscle subnetworks ChestL-ChestR and ArmL-ArmR, comparing the Beginning and End segments of Exercise 1. Boxplots correspond to the links strength stratification profiles in Fig. 3c, and represent dispersity of data from individual subjects with the effect of accumulated fatigue from Beginning to End of Exercise 1. **b** Boxplots representing subject statistics for the average links strength of the six modules (shown in different box color) for the subnetworks of all muscle pairs, comparing Exercise 1 with Exercise 2 and 3. Boxplots correspond to the links strength stratification profiles in Figs. 4c and 5b, and show data dispersity from individual subjects with the effect of residual fatigue for Exercise 2 and 3. Statistics support the presence of distinct links strength profiles that uniquely quantify each muscle pair subnetwork.

Notably, the uncovered reorganization in network interactions in response to residual fatigue across consecutive exercise bouts (Fig. 4 and Fig. 5) is consistent with the effect of fatigue accumulation during a single exercise bout on network structure (Fig. 3). Moreover, we find a similar modulation in the profile of network links strength for different subnetwork modules with accumulation of fatigue during Exercise 1 and with residual fatigue across Exercise 1, 2 and 3 (compare bar charts in Fig. 3c with bar charts in Fig. 4c and Fig. 5b). To provide a more comprehensive information about the distribution and variability of the data obtained from different subjects presented in the link strength profiles of the different subnetwork modules in Figs. 3c, 4c and 5b, we display the median links strength and interquartile range (boxplots) for all subnetworks with accumulation of fatigue during Exercise 1 (Beginning vs End segments; Fig. 6a), as well as with residual fatigue across consecutive exercise bouts (Fig. 6b).

### Patterns in intermuscular network links strength and their reorganization with residual fatigue

**Fig. 7:**
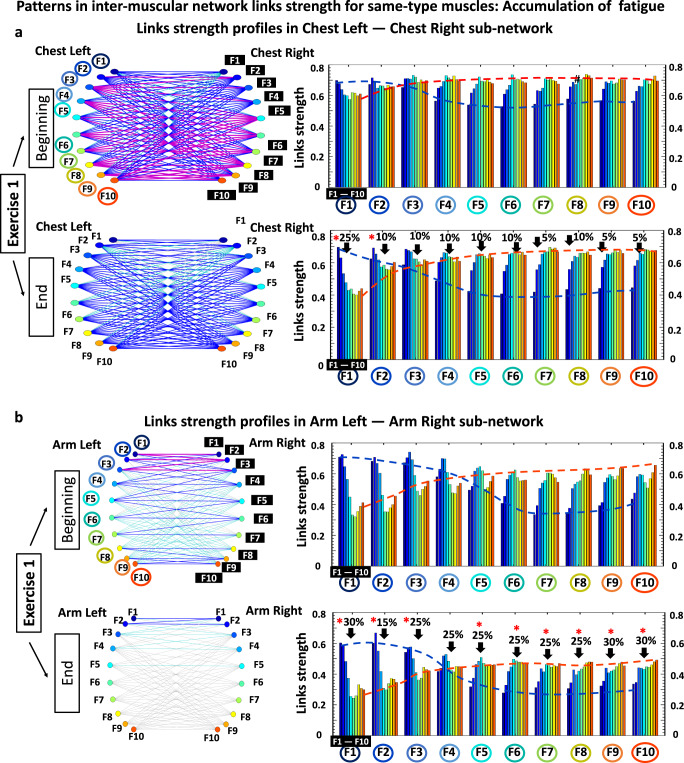
Profiles of links strength and network modules within same-type muscle subnetworks and their modulation in response to accumulation of fatigue during exercise. **a** Network interactions of frequency bands F_i_ (representing the activity of different muscle fiber types) within the ChestL-ChestR subnetwork. Subnetwork structure (left panels) and links strength profiles (right panels) of the ten basic network modules within the ChestL-ChestR subnetwork (each module represents the interaction of a given frequency band F_i_ from one muscle ChestL with all frequency bands from the other muscle ChestR) for the Beginning and End segments (top and bottom panels) of Exercise 1. Network structure and profiles of links strength dramatically reorganize with accumulation of fatigue during Exercise 1. Dynamic networks are derived from the group-averaged cross-correlation matrices for Exercise 1 in Fig. 2a, where network links correspond to the matrix elements and show the coupling strength (degree of synchronous activity; Fig. 1d) between distinct muscle fiber types from the ChestL and ChestR muscles (line color and width indicates network links strength; “Intermuscular interaction networks”, Methods). The ChestL-ChestR subnetwork topology is defined by the structure of the basic subnetwork modules — ten modules, with ten links each, form a complex hierarchical organization of links strength in the subnetwork which changes in response to accumulation of fatigue. Left panels: frequency bands F_i_ of the ChestL muscle are marked by circles and bands F_i_ of the ChestR muscle are marked by black squares. Same notation for F_i_ is used on the horizontal axis of the right panels with bar charts showing the link strength interaction profiles of the ten modules, where color bars within each profile correspond to the color of the network node (left panels) associated with a given frequency band in the Chest muscle. Red stars indicate statistically significant reduction in links strength for all modules comparing Beginning vs End of Exercise 1. Modulation of the profiles is marked by dash lines in color. **b** Same-type muscle ArmL-ArmR subnetwork (left panels) and links strength profiles for the ten subnetwork modules (right panels) with same notations as in (**a**). Modules in the ArmL-ArmR subnetwork exhibit similar links strength profiles as modules in the ChestL-ChestR subnetwork, however, with (i) weaker links and significantly higher stratification of links strength within each module for the Beginning segment of Exercise 1 (top right panel), (ii) more pronounced decline in links strength with fatigue accumulation for the End segment (bottom right panel), and (iii) preserved degree of links strength stratification within profiles comparing Beginning vs End (in contrast to the ChestL-ChestR subnetwork which shows less decline in link strength but a dramatic increase in profile stratification with accumulation of fatigue).

**Fig. 8:**
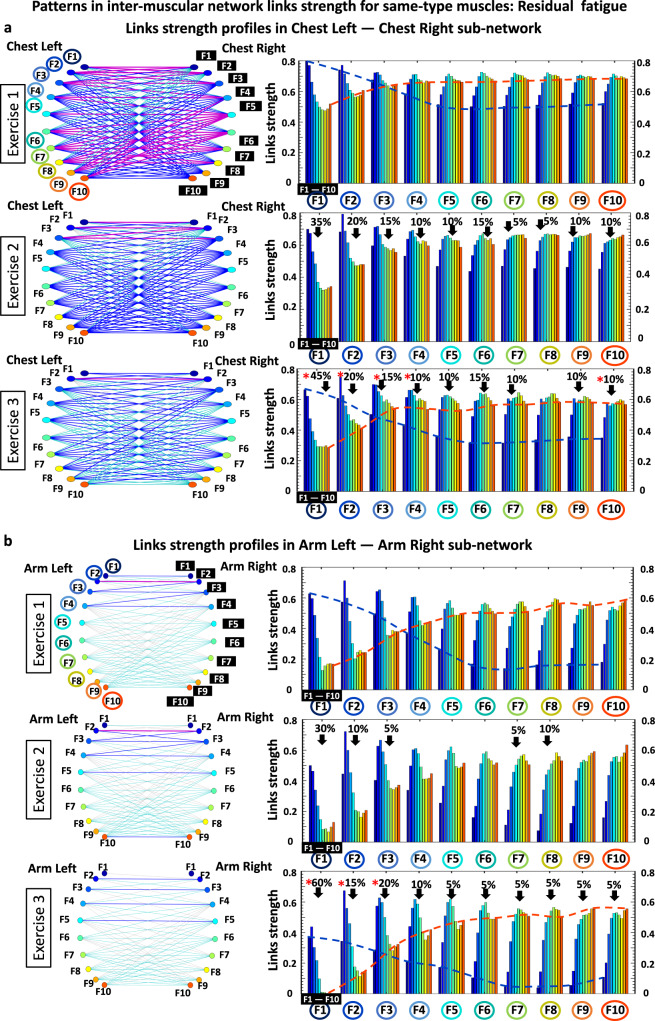
Profiles of links strength and network modules within same-type muscle subnetworks. **a** Network interactions of frequency bands Fi (representing the activity of different types of muscle fibers) within the ChestL-ChestR subnetwork. Links strength in the ten basic network modules within the subnetwork (each module represents the interaction of a given frequency band F_i_ from one muscle ChestL with all frequency bands from the other muscle ChestR) dramatically reorganize with fatigue for consecutive exercise bouts (left panels with networks). Dynamic networks are derived from the group-averaged cross-correlation matrices for Exercise 1, 2 and 3 in Fig. 2a, where network links correspond to the matrix elements and show the coupling strength (degree of synchronous activity) between distinct muscle fiber types from the ChestL and ChestR muscles (Line color and width indicates network links strength; “Intermuscular interaction networks”, Methods). The ChestL-ChestR subnetwork topology is defined by the structure of the basic subnetwork modules —ten modules, with ten links each, form a complex hierarchical organization of links strength in the subnetwork which changes in response to fatigue. Left panels: frequency bands F_i_ of the ChestL muscle are marked by circles and bands F_i_ of the ChestR muscle are marked by black squares. Same notation for Fi is used on the horizontal axis of the right panels with bar charts showing the link strength interaction profiles of the ten modules, where color bars within each profile correspond to the color of the network node (left panels) associated with a given frequency band in the Chest muscle. Red stars indicate statistically significant differences in links strength for all modules comparing Exercise 1 vs Exercise 3 (Wilcoxon test p values with multiple tests Bonferroni correction < 0.025). Modulation of the profiles is marked by dash lines in color. **b** Same-type muscle ArmL-ArmR subnetwork (left panels) and links strength profiles for the ten subnetwork modules (right panels) with same notations as in (**a**). Modules in the ArmL-ArmR subnetwork exhibit similar links strength profiles as modules in the ChestL-ChestR subnetwork, however, with significantly higher stratification of links strength within each module, less pronounced decline in links strength with fatigue, and preserved degree of links strength stratification within profiles for all exercise bouts.

**Fig. 9:**
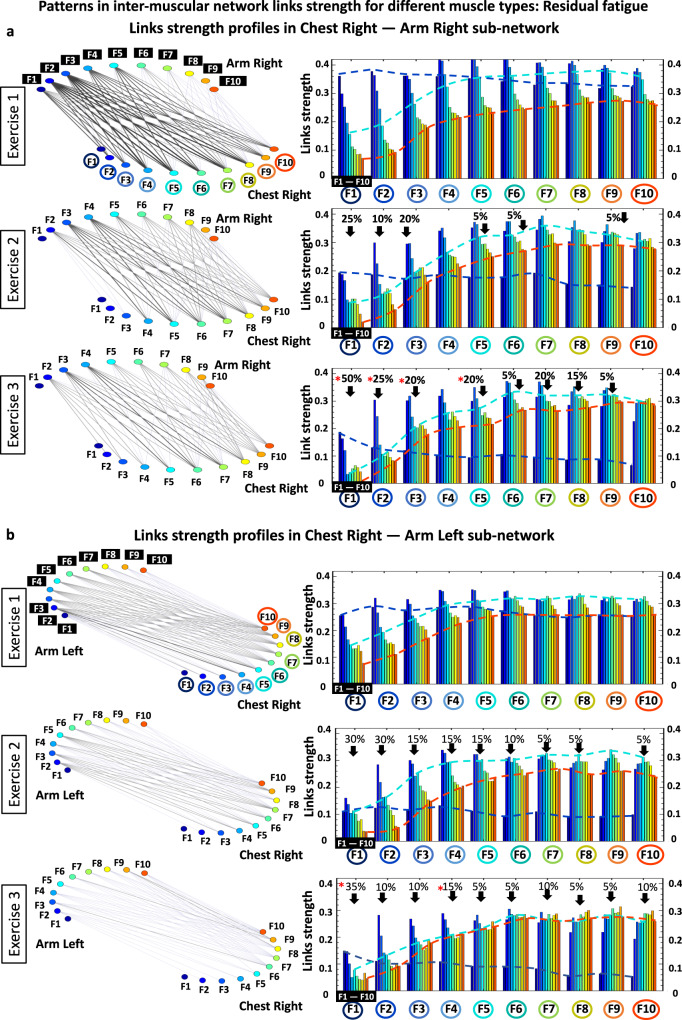
Profiles of links strength and network modules within different-type muscle subnetworks. **a** Interaction networks for myoelectrical rhythms within the ChestR-ArmR subnetwork where links strength represents the degree of synchronous activation in the spectral power of distinct muscle fibers working in different frequency bands F_i_. During Exercise 1, the ChestR-ArmR subnetwork topology is characterized by a hierarchical organization of ten basic modules, where each module represents the interaction of a given frequency band F_i_ from ChestR with all frequency bands from ArmR in the subnetwork. Right panels: frequency bands F_i_ for the ChestR muscle are marked by circles on the horizontal axis of each panel, and for the ArmR muscle are marked by black squares within each subnetwork module. Bars color in each module profile corresponds to the color of the node (left panels) associated with a given frequency band in the muscle. Red stars indicate statistically significant differences in links strength for all modules comparing Exercise 3 vs Exercise 1 (Wilcoxon test p values with multiple tests Bonferroni correction < 0.025). Color dash lines mark changes in the profile for the ten modules. **b** Same network representation and links strength profiles of subnetwork modules as in (**a**) are shown for the different-type muscle ChestR-ArmL subnetwork. Modules in the ChestR-ArmL subnetwork exhibit (i) similar shape for their links strength profiles as the modules in ChestR-ArmR subnetwork; (ii) similar modulation of the profiles shape with accumulation of fatigue during Exercise 2 and 3.

The results shown in Figs. 4 and 5 reveal complex dynamic patterns of muscle fiber synchronous activity, and a structured hierarchical organization of distinct network modules and subnetworks. The stratification profiles of average links strength within separate subnetworks and network modules, however, show a course-grained level of network organization and dynamics in response to fatigue. To probe the fine structure within the hierarchical organization of the global intermuscular network, we analyze all individual links for all modules in each subnetwork: two same-type subnetworks (ChestL-ChestR and ArmkL-ArmR, Figs. 7 and 8), and four different-type subnetworks (ChestR-ArmR, ChestR-ArmL, ChestL-ArmL and ChestL-ArmR, Fig. 9). We observe that links within individual modules (10 links per module representing the degree of coupling between a frequency band F_i_ from one muscle with all frequency bands of the other muscle in a given subnetwork, see Methods) exhibit unique profiles specific for each subnetwork (10 modules and 100 links per subnetwork, Methods). This represents a marker of the function that different subnetworks play in coordinating interactions among all muscles in the global network.

#### Same-type muscle subnetworks

The ChestL-ChestR subnetwork show complex interactions among frequency bands F_i_ representing distinct types of muscle fibers (Fig. 8a, right panels). Specifically, during Exercise 1 a profile with declining links strength characterizes the subnetwork module of interaction between F1 frequency band of ChestL with all ten frequency bands of ChestR (first group of ten bars in Fig. 8a; top right panel). This contrasts with the modules F9 and F10 characterized by increasing links strength, while modules F3 and F4 show flatter profiles during Exercise 1. Further, during Exercise 2 and Exercise 3 the strength of all links significantly declines across all ten modules in the ChestL-ChestR subnetwork, while the stratification in the links strength profile of each subnetwork module increases with fatigue (higher separation between first and last bar in each group of 10 bars; higher separation between blue and red dotted lines in Exercise 3 vs Exercise 1 in Fig. 8a).

Regarding the modules in the ArmL-ArmR subnetwork, we observe a similar shape for their links strength profiles as modules in the ChestL-ChestR subnetwork, however, with higher stratification of links strength within each module as well as less pronounced decline in links strength during Exercise 2 and Exercise 3 (Fig. 8b right panels). Remarkably, in contrast to the ChestL-ChestR subnetwork, the degree of links strength stratification of the profile for each module within the ArmL-ArmR subnetwork does not significantly change with repeated exercise bouts (similar separation between first and last bar in each group of 10 bars / similar separation between blue and red dotted lines in Exercise 3 vs Exercise 1). These empirical observations reveal that same-type muscle subnetworks, which are the major contributors to the push-up movement, exhibit similar hierarchical organization (profiles of links strength) within subnetwork modules, while the links strength stratification within each module and the degree of decline of links strength in response to fatigue is markedly different. This reflects physiological differences related to the different role of the two subnetworks during squat movements — i.e., primary dynamic role of the ChestL-ChestR subnetwork vs secondary role of the ArmL-ArmR subnetwork.

#### Subnetworks of different muscle type

The topology of links strength in the ChestR-ArmR subnetwork (muscles on the same side of the body) reorganizes with fatigue for consecutive exercise bouts (Fig. 9a, left panels). Specifically, during Exercise 1 links within the ChestR-LegR modules form a similar profile with declining strength — i.e., the degree of coupling for all frequency bands in the ChestR muscle declines for increasing frequency bands in the ArmR muscle (Fig. 9a, top right panel). During Exercise 2 and Exercise 3, the shape of the links strength profiles for all modules in the ChestR-ArmR subnetwork gradually changes with progression of fatigue, where the interactions of all frequency bands in the ChestR muscle with the F1 and F2 bands of the ArmR muscle (first two bars in each module of ten bars) significantly decline (dashed dark blue guiding line, Fig. 9a right panels), while the strength of interactions with the intermediate and high frequency bands in the ArmR muscle remains the same (dashed light blue and red guiding lines, Fig. 9a right panels).

Regarding the modules in the ChestR-ArmL subnetwork (Fig. 9b right panels; muscles on the opposite side of the body), we find a (i) similar shape for their links strength profiles as the modules in the ChestR-ArmR subnetwork (Fig. 9a); and (ii) similar modulation of the profiles shape with accumulation of fatigue for Exercise 2 and 3. The stratification of links strength profiles in the modules of both different-type muscle subnetworks ChestR-ArmR and ChestR-ArmL progressive decreases with fatigue for repeated Exercise 2 and Exercise 3. We observe a similar network structure and evolution of links strength profiles during Exercise 2 and Exercise 3 for the other two different muscle types ChestL-ArmL and ChestL-ArmR subnetworks (matrices in Fig. 2a).

## Discussion

The present study investigates how distinct muscle fiber types dynamically synchronize with each other and integrate as a network across several chest and arm muscles during a maximal (till exhaustion) push-up test, and how the network of intermuscular interactions reorganizes with accumulation of fatigue during consecutive exercise bouts. Uncovering the nature of network interactions among muscle fibers is key to (i) understanding the mechanisms regulating the function of individual muscles and (ii) how muscles coordinate to facilitate variety of movements. One possible hypothesis is that each fundamental movement pattern (e.g., push-up, squats, etc) is associated with a unique network organization, with specific patterns of cross-frequency communication among muscle fibers across muscles and specific response to exercise-induced fatigue. An alternative hypothesis is that different movement patterns are characterized by an intermuscular network with universal hierarchical organization of subnetworks and modules, that does not depend on the diversity of the muscles involved in a particular movement pattern but rather reflects the role distinct muscles play in facilitating the movement. Remarkably, the reported here empirical results confirm the second hypothesis, since the uncovered network structure representing coordination between chest and arm muscles for the maximal push-up test is consistent with the network organization of cross-frequency communication between leg and back muscles reported recently for maximal squat test^24^. Thus, our findings indicate the existence of general physiological principles of intermuscular coordination for different fundamental movement patterns, so that muscle network interactions and their reorganization with fatigue do not depend on the specific movement but rather reflect the role that pairs of muscles play during each movement. Further empirical and modelling investigations would be needed to confirm the validity of such general principles.

Specifically, this study provides empirical evidence that different-type muscle fibers synchronize their activity across muscles by integrating into distinct dynamic patterns of cross-frequency networks during push-up movement. We demonstrate that (i) pairs of muscles in the global intermuscular network form subnetworks with specific signatures of cross-frequency communication to synchronize activation among muscle fibers, and (ii) each subnetwork consists of multiple modules with specific profiles of links strength stratification that follow different evolution paths in response to accumulation of fatigue.

During Exercise 1 in our test protocol, the same-type muscle ChestL-ChestR subnetwork exhibits unique hierarchical organization with a specific stratification profile formed by the average links strength of the distinct modules within the subnetwork. Network modules representing the interactions of low-low ([F1-F2]—[F1-F2]), intermediate-intermediate ([F3-F7]-[F3-F7]), intermediate-high ([F3-F7]-[F8-10]) and high-high ([F8-F10]—[F8-F10]) frequency bands exhibit dominant links strength, while modules corresponding to low-intermediate ([F1-F2]—[F3-F7]) and low-high ([F1-F2]—[F8-F10]) frequency bands show moderate links strength (Fig. 4c). Since the average conduction velocity of an active motor unit relates to the muscle fiber type^4^, and changes in EMG spectral properties are linked to changes in the average conduction velocity of motor units^5,6^, the uncovered links strength stratification profile in the ChestL-ChestR subnetwork during Exercise 1, indicates that slow type-I muscle fibers in the ChestL muscle predominantly coordinate (synchronize activation) with slow type-I fibers in the ChestR muscle, while intermediate type IIa fibers coordinate with both type IIa and fast type IIb fibers. This finding reflects that muscle fibers from different muscles synchronize better when they have similar histochemical characteristics, and thus, subnetwork modules corresponding to low-low, intermediate-intermediate and high-high frequency band interactions show stronger links (higher bars in the profile shown in Fig. 4c).

Notably, the characteristic links strength stratification profile observed in the ChestL-ChestR subnetwork during Exercise 1 is also present for the other same-type muscle ArmL-ArmR subnetwork, however, with remarkably lower coupling strength for low-intermediate and low-high frequency band interactions compared to the ChestL-ChestR subnetwork. This indicates that, unlike the ChestL-ChestR subnetwork, slow type-I muscle fibers in the ArmL muscle cannot communicate with intermediate type IIa and fast IIb muscle fibers in the ArmR muscle, leading to a links strength profile with higher initial stratification (spread) during Exercise 1. The uncovered differences between the ChestL-ChestR and ArmL-ArmR subnetworks may reflect the distinct role these subnetworks play in push-up movements — while pectoralis major (Chest) have primary motor role during push-up movements (higher myoelectrical activation), triceps brachii (Arm) has a secondary role^38,39^. Interestingly, the modules with strongest links in both same-type muscle subnetworks ChestL-ChestR and ArmL-ArmR are those that correspond to the low-low ([F1-F2]—[F1-F2]) frequency bands interactions, even though subnetworks play different role in push-up movements. This finding agrees with a recent study showing strong intermuscular coherence in the alpha and lower beta rhythms during walking^40^, which fall within the range of F1-F2 frequency bands in our study. It has been previously shown that cortical beta-band activity is linked with specific patterns of distributed muscle activity (muscle synergies), which are involved in controlling forces to decelerate and accelerate the body^41–43^, and thus, could lead to the high degree of synchronous muscle activation in the low-frequency bands observed for the same-type muscle subnetworks in our study.

The links strength stratification profile for the modules in the ChestL-ChestR subnetwork during Exercise 1 is consistently observed also for Exercise 2 and Exercise 3, however, with (i) significantly reduced coupling strength and (ii) increased stratification (spread) due to a more pronounced reduction in average links strength for the subnetwork modules representing coupling of the low-high frequency bands (Fig. 4c). These finding demonstrate that fatigue provokes a decrease in the cross-frequency coordination among distinct type of muscle fibers within the ChestL-ChestR subnetwork, while strongest interactions are mediated by the same type of muscle fibers.

Similarly to the ChestL-ChestR subnetwork (major role in push-ups), the general shape of the links strength profile observed for Exercise 1 in the ArmL-ArmR subnetwork (secondary role in push-ups) does not change with accumulation of fatigue during Exercise 2 and Exercise 3, and both subnetworks exhibit reduction in links strength with fatigue (Fig. 4c). However, for the ArmL-ArmR subnetwork we find a less pronounced stratification (spread) of the links strength profile with fatigue due to a similar decrease of links strength for the low-low ([F1-F2]—[F1-F2]), low-intermediate ([F1-F2]—[-[F3-F7]), and for low-high ([F1-F2]—[F8-F10]) modules (Fig. 8b). The observed here decrease of network links strength during Exercise 2 and 3 for both ChestL-ChestR and ArmL-ArmR subnetworks could be explained by the loss of neuromuscular complexity typically associated with neuro-muscular fatigue^44^. The impact of fatigue above the critical torque (severe exercise demands) is not limited to force-generating capacity. It has been conjectured that the impact of fatigue extends to the adaptability of the neuromuscular system to external perturbation^45^, and our empirical results confirm this conjecture demonstrating loss of network coordination among muscle fibers in the neuromuscular system represented by significant decline in network links strength. Notably, since common synaptic input increases in response to fatigue, there is an increment in common oscillations of motor neuron discharge rates, which could lead to a decreased level of complexity (increased regularity)^46^ and corresponding increase in iso-frequency coherence^12,14,15^. In contrast, our comprehensive cross-frequency network approach demonstrates more intricate behavior, where the coordinated activity (links strength) among muscle fibers of different-type across muscles may decline or increase with fatigue for different modules within the subnetworks (Figs. 3c, 4c and 8).

Notably, we find that accumulation of fatigue within an exercise bout and residual fatigue across exercise bouts lead to a similar response with modulation of links strength and reorganization in network structure (compare results shown in Fig. 3 vs Figs. 4–5, and in Fig. 7 vs Figs. 8–9). In summary, while there are certain quantitative differences associated with accumulation and residual fatigue, qualitatively the response to both types of fatigue in the networks of intermuscular coordination is characterized by a similar tendency.

Remarkably, the links strength stratification profile for the same-type muscle subnetworks (ChestL-ChestR and ArmL-ArmR) and its response to fatigue during the push-up, are consistent with the recently observed network organization for same-type muscle subnetworks (LegL-LegR and BackL-BackR) during a maximal squat test (3 consecutive bouts of squat exercise, each performed till exhaustion^24^. Note that ChestL-ChestR subnetwork during push-ups could be compared to LegL-LegR subnetwork during squats (both play primary role), while ArmL-ArmR during push-ups may be contrasted to BackL-BackR duirng squats (both play secondary/supportive role). Further, both ChestL-ChestR and LegL-LegR subnetworks exhibit the same links strength profile during Exercise 1, with dominant interaction for low-low, intermediate-intermediate and high-high frequency bands. In response to fatigue across repeated exercise bouts, both ChestL-ChestR and LegL-LegR subnetworks reduce links strength and increase profile stratification. In contrast, both subnetworks ArmL-ArmR and BackL-BackR present an initial links strength profile with moderate coupling and high stratification level during Exercise 1, and reduced links strength and no change in stratification with accumulation of fatigue for Exercise 2 and 3.

Regarding the different-type muscle ChestR-ArmR and ChestR-ArmL subnetworks, we observe a distinct profile of network links strength with remarkably weaker coupling strength during Exercise 1 (Fig. 2b, Fig. 5b), compared to the same-type muscle subnetworks (Fig. 4c; 8a). ChestR-ArmR and ChestR-ArmL intermuscular interactions are predominantly mediated by low-low and intermediate-intermediate frequency bands. This reflects that slow type-I and intermediate type IIa fibers in the chest muscle synchronize activity mainly with the matching type-I and IIa fibers in the arm muscle. This finding agrees with a recent work showing lower intermuscular coherence between distant muscles in different legs compared to topologically closer muscles during walking^40^. Similarly to same-type muscle ChestL-ChestR and ArmL-ArmR subnetworks (Fig. 4c, Fig. 8), links strength profiles of the different-type muscle ChestR-ArmR and ChestR-ArmL subnetworks during Exercise 2 and 3 are characterized by a decrease in average link strength with fatigue accumulation, however, with a decline in the stratification (spread) of the interaction profiles due to targeted decrease of links strength only for the modules representing low-low, low-intermediate, and intermediate- intermediate frequency bands interactions (Fig. 5b, Fig. 9).

Importantly, the links strength stratification profile for the different muscle type subnetworks (ChestR-ArmR and ChestR-ArmL) during push-ups, is consistent with the recently uncovered network organization for different muscle type subnetworks (LegR-BackR and LegR-BackL) during the squat movement^24^, with dominant interactions for low-low and intermediate-intermediate frequency bands. However, the response to fatigue with repeated Exercise 2 and 3 is markedly different. While ChestR-ArmR and ChestR-ArmL subnetworks (push-ups) gradually decrease coupling strength (Fig. 9), LegR-BackR and LegR-BackL (squats) increase the degree of synchronization (Fig. 6 in^24^). These differences reflect distinct histochemical composition and function between Back (erector spinae) and Arm muscles (triceps brachii). Erector spinae is a fatigue-resistant muscle with predominantly slow type-I muscle fibers^47^ which plays a stabilization/supportive role during squats, while the triceps brachii has higher composition of fast type-II muscle fibers^48,49^ and presents a more dynamic role during push-ups associated with gradual decline in links strength with fatigue. These findings demonstrate high sensitivity of our method to assess and quantify intermuscular coordination.

Our approach to quantify intermuscular network interactions of different types muscle fibers is motivated by empirical observations of synchronous changes in the spectral power of EMG frequency bands that reflect spontaneous activation of different muscle fiber types (Fig. 1d). Thus, naturally in this pilot study we analyzed the degree of cross-correlation between the EMG spectral power time series for different frequency bands (amplitude-amplitude coupling; Fig. 1d), where we first normalized the data to zero mean and unit standard deviation to reduce spurious effects of trends (Methods Section “Spectral decomposition ”)^50,51^. Alternative methods such as phase synchronization^52–54^, phase-amplitude coupling^55–57^ and time-delay stability^30,58,59^ could be employed to probe various aspects of the coupling dynamics and network interactions among muscles fibers. Indeed, recent studies have demonstrated the presence of several independent forms of interaction between key physiological systems — e.g., the cardiac and respiratory systems communicate through three coexisting forms of interaction that reflect different aspects of autonomic regulation^60,61^. It is plausible that the intermuscular coordination in bursting activity of different muscle fiber types is also mediated by several distinct forms of interaction (in addition to the reported here amplitude-amplitude cross-correlation) to facilitate a variety of fine movements and behaviors — hypothesis that will be investigated in future studies.

Our findings of complex hierarchically-structured network interactions across topologically distant chest and arm muscles align with the recently proposed framework on motor control and muscle coordination^21^, postulating that the central nervous system does not control muscles, but rather controls specific functional clusters composed of motor neurons within and across muscles to facilitate given movement. This framework is supported by empirical demonstrations that motor neurons from distant muscles receive common neuronal input during exercise (e.g.,^41^). This suggests a possible control mechanism where a fraction of motor neurons within a pool that innervates a given muscle interact with a fraction of the motor neurons that innervate another muscle, thus, forming a distinct functional cluster associated with a given movement, and that multiple functional clusters of motor neurons with differentiated role across distant muscles facilitate a broad range of movements. In other words, each motor unit may generate a force to assist movement in particular direction and, thus, the recruitment of specific groups of motor neurons across several muscles may be a strategy to comply with the mechanical demands of a given movement task. The uncovered here distinct modules within subnetworks, in fact represent distinct functional clusters that allow synchronization of activity for distant chest and arm muscles during push-ups.

**Fig. 10:**
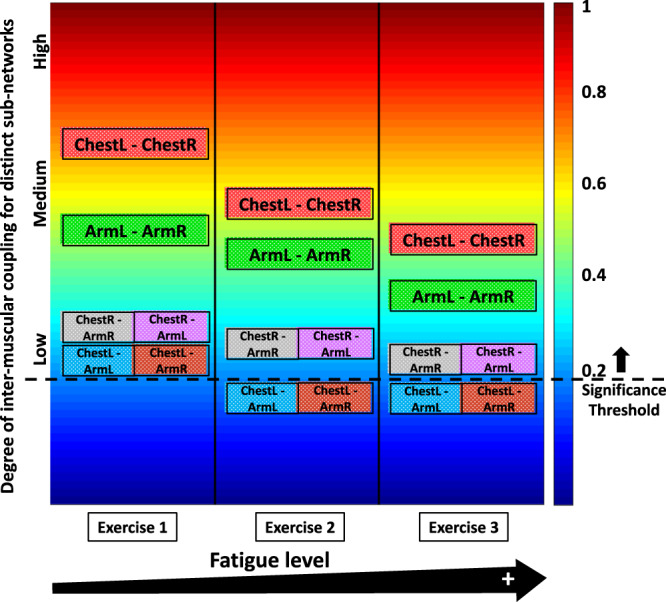
Degree of intermuscular coupling for the subnetworks involved in push-up movement, and change in the strength of network interactions with fatigue. During Exercise 1, the average links strength for the same-type muscle subnetworks ChestL-ChestR and ArmL-ArmR exhibit intermediate to high degree of coupling. Note that ArmL-ArmR interactions are weaker than ChestL-ChestR. In contrast, the different-type muscle subnetworks ChestR-ArmR, ChestR-ArmL, ChestL-ArmL and ChestR-ArmR present low degree of coupling, indicating less synchronous activation of distinct types muscle fibers across these muscles. Further, this reflects distinct link strength profiles of subnetwork interactions that play role in coordinating muscle fibers activity between different-type muscles (Fig. 9) compared to the same-type muscle subnetworks (Fig. 8). With accumulation of fatigue during Exercise 2 and 3, interactions in the ChestL-ChestR and ArmL-ArmR subnetworks become remarkably weaker as these subnetworks account for the major dynamic effort in generating push-up movement, and are quickly affected by fatigue. Similarly, coupling strength for the different-type muscle Chest-Arm subnetworks is reduced with fatigue, however, the reduction is less pronounced compared to same-type muscle ChestL-ChestR and ArmL-ArmR subnetworks. Notably, Chest-Arm interactions on the right side of the body (ChestR-ArmR, ChestR-ArmL) are stronger than interactions in left side (ChestL-ArmL, ChestR-ArmR) since all participants in this study were right-handed.

In summary, this study focuses on understanding the dynamics of intermuscular interactions through patterns of synchronous rhythms embedded in myoelectrical activation, and how theses rhythms integrate as a network to coordinate the activity of different-type muscle fibers across distinct muscles to facilitate targeted movements. Specifically, we consider a maximal push-up test of repeated exercise bouts to explore the effect of accumulated fatigue on the dynamics of synchronous myoelectrical activity coordination, and on the hierarchical organization of the underlying network structure. We find that different-type muscle fibers within the chest and arm muscles (involved in the push-up movement) exhibit synchronous activity characterized by cross-frequency patterns of coordination that depend on the role of muscles involved in the movement. Our analyses show that the global network with complex hierarchical organization, consisting of distinct subnetworks and network modules, integrate muscle fibers from different muscles and underlie their coordination during the movement. Notably, each pair of muscles in the global intermuscular network forms a distinct subnetwork which (i) depends on the role (primary, secondary) the muscles play in the push-up movement, (ii) is characterized by specific pattern in network links strength representing cross-frequency communication among muscle fibers, and (iii) undergoes a particular phase-space trajectory in connectivity and coupling strength with fatigue for repeated exercise (Fig. 10). Further, each subnetwork is structured by multiple modules with unique profile of links strength representing the degree of synchronization among muscle fibers. Moreover, we establish how the global intermuscular network changes during consecutive push-up bouts in response to fatigue. Our empirical analyses show that subnetworks for pairs of same-type muscles have different organization compared to subnetworks of different-type muscles, and that subnetwork organization strongly depends on the role of the muscles in the push-up movement. In particular, very complex links strength organization with pronounced stratification is observed for the same-type muscle subnetworks (primary motor muscle pairs of ChestL-ChestR and ArmL-ArmR), while subnetworks of different-type muscle (secondary muscle pairs of Chest-Arm) exhibit different links strength profile with different response to fatigue.

Importantly, our findings demonstrate that the recently uncovered profiles of cross-frequency links strength between leg and back muscles during squat movements^24^ are also present in the network interactions between chest and arm muscles during push-up movements. This indicates that while squats and push-ups (i.e., different movement patterns) involve distinct muscles and movement biomechanics, the underlying network of intermuscular interactions are characterized by general principles of hierarchical organization (subnetworks and modules) that do not depend on the diversity of the muscles involved, but rather represent the role of the muscles during the movement (e.g., primary, secondary or compensatory role). Thus, our empirical observations support the hypothesis that fundamental movement patterns of the human organism (squat, push, pull, hinge etc.) involving various combinations of muscles and micro-movements might be represented by just a few classes of intermuscular interaction networks, where each class is associated with specific hierarchical organization. Further research is needed to investigate how distinct muscle fibers dynamically synchronize their activation across muscles and respond to fatigue during other fundamental movement patterns.

We note that what we study here is an adaptive network of dynamic systems (muscle fibers represented by EMG frequency bands) which continuously interact and coordinate activity through neurophysiological processes^62^. In such adaptive network a change in the dynamics and coordination of some network nodes (muscle fibers) can lead to adaptive responses through changes in the dynamics and interaction of other nodes. Thus, while the functional networks uncovered here characterize intermuscular coordination among muscle fibers with different histochemical characteristics, the hierarchical structure and global dynamics of the networks can facilitate the coordination of activity and interaction strength of particular muscle fibers (dynamic nodes in the network) within and across muscles. Based on this reasoning, further investigations would be needed within the theoretical framework of adaptive networks to fully understand the underlying regulatory processes of intermuscular muscle fiber coordination.

The Network Physiology framework^36,37^ we employ here leads to new insights on the mechanisms regulating intermuscular interactions, and can have broad implications for diverse exercise-related phenomena, including sports performance, fatigue, overtraining or muscle-skeletal injuries. From a practical point of view, our approach can be utilized to develop a new set of network-based biomarkers able to assess and quantify intermuscular interactions during distinct fundamental movement patterns. Establishing the default intermuscular network for each movement pattern (e.g., squat, horizontal and vertical push or pull) as well as the healthy network reorganization in response to fatigue, will be of key relevance to complement and overcome the limitations of the commonly utilized physiological parameters to assess health and human performance, and to diagnose neuro-degenerative disorders. Notably, gold standard physiological and performance variables (e.g., power, VO2max etc.) provide little information on the nonlinear dynamic interactions among physiological systems^27,37,59^, and cannot inform about the qualitative synergetic reconfigurations that distinct physiological systems undertake to adjust the individual response to exercise requirements^63–66^. Accordingly, more research is needed to (i) confirm the universality of our results over larger cohorts of subjects and identify the reference intermuscular network profiles for all basic movement patterns (e.g., squat, horizontal and vertical push or pull etc.); (ii) study the processes of reorganization and breakdown of intermuscular network interactions under various clinical conditions (e.g., acute and chronic muscle injuries, neuro-muscular and neuro-degenerative disorders, etc.), and (iii) investigate synchronized cross-frequency communication among different muscles and key organ systems, such as the heart or the brain and related cortical rhythms^30,67,68^. Extending investigations in these directions would lay down the first building blocks of a new interdisciplinary area of research, Network Physiology of Exercise^69–71^.

## Methods

### Subjects

Eighteen healthy young male adults (age 24.87 ± 2.14 years, height 174.65 ± 10.09 cm, and mean body mass 74.88 ± 8.34 kg) were recruited for this study. Participants were strictly recruited according to the following inclusion criteria: a) aged 20-30 years; b) BMI (in kg/m^2^) > 18.5 and < 30; c) normal physical activity > 5 and < 10 h/week, but without sport specialization (not active athletes), and d) blood pressure < 140/90 mmHg. The following exclusion criteria were applied: a) intake of prescribed drugs that could negatively affect muscle strength, such as corticosteroids; b) no current or previous injury that could prevent performance during the experimental protocol test, and c) any other physical condition (cardiac, respiratory etc.) that may have prevented the performance of a test protocol involving push-up exercise until exhaustion. The experimental protocol was approved by the local ethical committee (Clinical Research Ethics Committee of the Sports Administration of Catalonia), and was carried out according to the Helsinki Declaration. Before taking part in the study, participants read the study description and risks, and signed an informed consent. All ethical regulations were followed. To determine the sample size for this study, a power analysis was conducted using G-Power 3.1. Previous studies assessing fatigue effects on repeated exercise performed until exhaustion (same research design; 1 group, 2–3 repeated measures) have reported large effect sizes (0.8 and higher; Garcia-Retortillo et al., 2019). Thus, using an effect size of 0.8, α < 0.05, power (1 − β)  =  0.80, we estimated a minimum sample size = 15.

### Study design and test protocol

After obtaining signed consent, participants visited the laboratory only one time. First, participants were be familiarized with the push-up movement — they practiced until they were able to execute the movement according to the protocol instructions (see study test protocol below). After at least a 5-minute rest, participants performed the study test protocol consisting of 3 sets of push-ups until exhaustion (Exercise 1, Exercise 2 and Exercise 3), interspersed by 5-min resting periods. The push-up sets will be performed according to the following instructions: 1) participants lied down (prone) with their knees on the floor and the hands on the preset marks (inter-acromial distance); 2) they did full elbow extension and place the arms perpendicular to the floor; 3) then, they lowered their bodies (straight line from shoulders to the knees) until the sternum was at 3 cm from the floor where a rope was placed; 4) when the rope was touched, they returned to standing position, and 5) repeated the push-up movement until exhaustion (Fig. 1a, b)^31–33^. The push-ups pace was controlled by a metronome (Metronome Beats, Stonekick, UK), using a 3:3 tempo with three seconds down movement and 3 seconds up. Thus, one single push-up lasts 6 seconds. Each push-up set finished when participants were not able to do the next push-up or, alternatively, when they could not maintain the required 3:3 tempo. This means, that the observed changes in the intermuscular networks of muscle fiber interactions (measured by cross-frequency coupling) are induced by fatigue (both accumulated during an exercise bout, and residual across exercise bouts) provoked by the push-up movement (Figs. 3–5).

The repetition of three consecutive exercise bouts performed until exhaustion with short 5-min resting period between bouts allows to identify the effects of: (i) acute fatigue (accumulation of fatigue within exercise segments), and (ii) residual fatigue across Exercise 1, Exercise 2 and Exercise 3. Whereas acute fatigue occurs when the energy consumption exceeds the muscle aerobic capacity and a large fraction of the required energy has to come from anaerobic metabolism^72^ residual fatigue is characterized by neuromechanical and biochemical alterations (e.g., decrease in maximal force) provoked by previous exercise (i.e., Exercise 1 and Exercise 2)^73^.

Two markers were utilized to assess the levels of fatigue and global physiological function in this manuscript. The first one is the amplitude of the EMG signal. Henneman’s Size Principle states that motor units within a motor unit pool are recruited in an orderly manner, based on their excitability, from the smallest to the largest unit^74,75^. This means that slow type-I, low-force, fatigue-resistant muscle fibers are activated before fast type-II, high-force, less fatigue-resistant muscle fibers. As fatigue increases, more motor units need to be activated and recruited to maintain the same force output during push-ups. Consequently, surface electromyography (sEMG) amplitude increases as more motor units are recruited to maintain force output^76^. As show in Fig. 1c, the amplitude of the EMG signal in both chest and arm muscles increases (i) within Exercise 1 (accumulation of fatigue) and across consecutive exercise bouts (residual fatigue; note the higher EMG amplitude at the beginning of Exercise 3 compared to Exercise 1). Same behavior is observed for the spectral power time series of individual muscle fiber types (frequency bands) where spectral power gradually increases with progression of the exercise bout — three-to-five-fold increase in spectral power amplitude comparing the Beginning (no fatigue) and the End (high fatigue) of the exercise bout (Fig. 1d).

### Electromyography acquisition and EMG signal processing

Participants were asked to wear appropriate clothing that would not obstruct EMG electrode placement sites. Before the mounting of the EMG electrodes, participants’ skin was shaved and cleaned using alcohol, and left to dry for 60 s to reduce the myoelectrical impedance, according to the SENIAM guidelines^77^. EMG signals from the following muscles were recorded simultaneously during the entire exercise protocol: left and right pectoralis major (Chest-Left and Chest-Right); left and right triceps brachii lateral head (Arm-Left and Arm-Right). The location of the surface electrodes (pre-gelled Ag/AgCl bipolar surface electrodes, DORMO LF-50) on each muscle was also carried out according to the recommendations of SENIAM organization. More specifically, pectoralis major electrodes were placed at over the center of the muscle belly along in the principal direction of muscle fibers of the pectoralis major (sternocostal part),  halfway between the sternal notch and anterior axillary line, approximately 2 cm apart in-line with muscle fibers^78,79^, and the triceps brachii electrodes were placed at 50 % on the line between the posterior crista of the acromion and the olecranon at 2 finger widths medial to the line (Fig. 1a, b). After the electrodes were secured, a quality check was performed to ensure EMG signal validity. The aforementioned Chest and Arm muscles were selected since they present the highest myoelectrical activity during the push-up movement^38,39^.

Data were recorded using a mDurance system (mDurance Solutions S.L., Granada, Spain), which includes a portable sEMG system (Shimmer3 EMG unit, Realtime Technologies LGd, Dublin, Ireland^80^, and processed by means of Matlab (Mathworks, Natik, MA, USA). Raw data was recorded at a sample frequency of 1024 Hz and filtered online using a 5–250 Hz band-pass filter. A Notch filter was used with a width of 1 Hz at the frequency of 50 Hz (i.e., 49.5 – 50.5 Hz) to remove line interference from the grid. All EMG recordings were visually inspected and only signals without noise artifacts were utilized in the analysis.

Note that in this study we employ the same method to assess and quantify intermuscular interactions as in a previous work^24^ (i) spectral decomposition (2.4.), (ii) cross-correlations between time series of spectral power (2.5.), (iii) Fourier phase randomization surrogate test (2.6.), as well as (iv) same data visualization method (cross-correlation matrices and intermuscular interaction networks; 2.7. and 2.8.).

### Spectral decomposition

To uncover how distinct muscle fibers dynamically coordinate and synchronize their activation and integrate as a network across different muscles during the push-up movement, we first segment the EMG signals from the left and right pectoralis major (Chest-Left and Chest-Right) and the left and right triceps brachii (Arm-Left and Arm-Right) into 2-sec non-overlapping time windows with 1-sec overlap across the three consecutive Exercise sets. Within each 2-sec time window, we extract the spectral power S(f) from each EMG signal using the ‘pwelch’ function in Matlab, based on the discrete Fourier transform (DTF) and the Welch’s overlapped segment averaging estimator. For each time window we obtain a spectral power value in bins of 0.5 Hz for the range [5–250 Hz], that is, *N* = 500 is the number of spectral power data points for each window of 2 seconds. To probe specific contributions from different frequency bands F_i_ to the spectral power within each 2-sec time window of the EMG signal, we consider 10 frequency bands with equal width of 19.5 Hz. These frequency bands correspond to the range of activity of different types of muscle fibers in each Chest and Arm muscle, i.e., F1 = [5-24.5 Hz], F2 = [25-44.5 Hz], F3 = [55-74.5 Hz], F4 = [75-94.5 Hz], F5 = [95-114.5 Hz], F6 = [115-134.5 Hz], F7 = [135-154.5 Hz], F8 = [155-174.5 Hz], F9 = [175-194.5 Hz] and F10 = [195-214.5 Hz].

We calculate the sum of the power $\widetilde{S}\left(f\right)$ across all frequency bins within each frequency band: $\widetilde{S}\left(f\right):=\mathop{\sum }\nolimits_{i=1}^{n}S({f}_{i})$, where ${f}_{i}$ are all n = 39 frequencies considered in each frequency band F_i_. Thus, we obtain 10 time series of EMG band power $\widetilde{S}\left(f\right)$ with 1-sec resolution for each muscle during the three exercise sets, representing the dynamics of all representative EMG frequency bands. Frequencies below 40-60 Hz (corresponding to our frequency bands F1, F2 and F3) could be attributed to the activity of small alpha-motor neurons that innervate type-I slow muscle fibers. The frequency range 60-120 Hz (bands F4, F5, F6 and F7) could be attributed to medium alpha-motor neurons that innervate type IIa intermediate (oxidative) fibers, and high frequencies in the range 170-220 Hz (bands F8, F9 and F10), correspond to large alpha-motor neurons that connect to type IIb fast (glycolytic) fibers^81–85^. The [45-54.5 Hz] range is filtered out by the Notch filter centered at 50 Hz to remove interference from electric grid, which modifies the EMG signal altering the spectral power of frequencies around 50 Hz. The obtained ten times series of EMG spectral power for each band F_i_ are then normalized to zero mean (µ = 0) and unit standard deviation (σ = 1). The obtained time series of EMG power $\widetilde{S}\left(f\right)$ in each frequency band F_i_ reflects the micro-architecture (in 1-sec resolution) of synchronous modulation in the amplitude of muscle activation, and allows to track variations in coupling and network interactions of EMG frequency bands F_i_ across different muscles with accumulation of fatigue (Fig. 1).

### Cross-correlations between time series of EMG spectral power in different frequency bands

To investigate cross-frequency interactions among muscle fibers (corresponding to EMG frequency bands), we consider all pairs of muscles involved in the push-up movement: (i) interactions of all frequency bands from same-type muscles (ChestL-ChestR and ArmL-ArmR) and (ii) different-type muscles (ChestL-ArmL, ChestL-ArmR, ChestR-ArmR and ChestR-ArmL). For each protocol set (Exercise 1, Exercise 2 and Exercise 3) and for each muscle pair, we calculate the bivariate equal-time Pearson’s cross-correlation for all pairs of time series representing EMG spectral power $\widetilde{S}\left(f\right)$ in the frequency bands F_i_ where i = 1,…,10. This leads to 10×10 = 100 cross-correlation values C_i,j_ for each pair of muscles, as shown in the intermuscular cross-correlation matrices (Fig. 2a) — i.e., C_i,j_ quantifies the degree of coupling EMG frequency band F_i_ from one muscle with the frequency band F_j_ from another muscle. The cross-correlation values range from C_i,j_ = −1 (fully anti-correlated) to C_i,j_ = 1 (fully positively correlated), with C_i,j_ = 0 indicating the absence of linear relation between the power $\widetilde{S}\left(f\right)$ time series of two EMG frequency bands. When cross-correlating the spectral power time series of two EMG frequency bands for an entire exercise bout, we found that the highest coefficient is the one corresponding to the zero lag (no delay). Our analyses of entire exercise bouts showed that the larger the delay in the cross-correlation, the lower the cross-correlation coefficient and thus, weaker interactions and network links.

### Fourier phase randomization surrogate test and significance threshold for links strength in networks of intermuscular interactions

To demonstrate the physiological significance of the networks of intermuscular interactions obtained from our empirical analysis, we perform a Fourier phase randomization surrogate test on the EMG signals recorded from the four muscles (ChestL, ChestR, ArmL and ArmR). The test preserves the global spectral power in the different frequency bands F_i_ within the EMG recording but destroys Fourier phase information related to nonlinear EMG characteristics. Thus, the test eliminates heterogeneities in the fine temporal structure associated with short time scale modulations in the spectral dynamics of EMG frequency bands F_i_ within a muscle, which reflect activation patterns of different muscle fiber types. Note that in the Fourier-phase-randomized surrogate EMG signals the relative ratios among the average spectral power of muscle activation in the frequency bands F_i_ are preserved, while synchronous modulations in EMG frequency bands that underlie effective cross-frequency coupling and account for the nonlinear characteristics of EMG signals are eliminated. As a result, the degree of coupling between EMG frequency bands F_i_ from different muscles is remarkably reduced after the Fourier Phase Randomization procedure, since physiologically relevant information regarding coordinated activation of distinct frequency bands is lost.

Further, to test the statistical significance and physiological relevance of the observed hierarchical network organization and its reorganization with fatigue, we perform an additional step in our surrogate test to establish the significance threshold for network links strength. Specifically, for each network link, surrogates are generated considering signals from each combination of two randomly chosen subjects. Since we have 18 subjects in the database, 153 pairs of random subject combinations are generated. Each muscle pair involves 100 links within the corresponding subnetwork between 10 frequency bands F_i_ (muscle fibers) of each muscle. Thus, combining the 6 subnetworks representing all muscle pairs we obtain a distribution of 54,600 surrogate links (cross-correlation values) for each protocol segment (Exercise 1, Exercise 2 and Exercise 3)— i.e., 6 muscle pairs x 100 links x 91 subject combinations = 91,800 surrogate links. For each protocol segment distribution the mean µ_*surr*_ and standard deviation σ_*surr*_ are obtained. Thus, the significance threshold at 95% confidence level for the network links strength is defined as µ_*surr*_ + 2*σ*_*surr*_ for each protocol segment. We find that the significance threshold for network links strength is Th_exercise_ = 0.2 (corresponding to the highest Th value during the exercise segments. The physiological significance threshold is represented by horizontal dashed black line in Fig. 2b.

### Intermuscular cross-correlation matrices

Group-averaged cross-correlation matrices represent pairwise coupling strength between the 10 frequency bands F_i_ of one muscle with the same bands derived from another muscle (i.e., 6 distinct muscle pairs: ChestL-ChestR, ChestL-ArmL, ChestL-ArmR, ChestR-ArmL, ChestR-ArmR and ArmL-ArmR) during a given protocol segment (Exercise 1, Exercise 2 and Exercise 3) (Fig. 2a). Matrix elements indicate the group-averaged (for all 18 subjects in the database) coupling strength between a frequency band that represents the activation of a specific muscle fiber type in a given muscle and a frequency band from another muscle. The dynamics of each muscle is represented by 10 EMG frequency bands with equal width of 19.5 Hz in the interval [5-215 Hz] (“Spectral decomposition”, Methods), and thus, each matrix consists of 100 elements (cross-correlation coefficients) representing interactions for each pair of muscles during a given Exercise set (Fig. 2a).

### Intermuscular interaction networks

A multiplex network of subnetworks is obtained to visualize interactions from all pairs of muscles and their hierarchical organization within the network (Fig. 3a. 4a). We map the group-averaged matrices in Fig. 2a into different networks for the Beginning (first third) and End (last third) segments during Exercise 1 (Fig. 3a; accumulation of fatigue), and across Exercise 1, Exercise 2 and Exercise 3 (Figs. 4 and 5; residual fatigue). The graphical approach we employ is essential to identify universal patterns in the intermuscular network structure, the hierarchical organization of subnetworks and modules, and to track the transition in network characteristics with accumulation of fatigue. Each muscle is represented by a semicircle where color nodes represent distinct frequency bands F_i_ corresponding to different muscle fiber types in the muscle (“Spectral decomposition ”, Methods). Network links correspond to the values of cross-correlation matrix elements C_i,j_ in Fig. 2a and reflect the coupling strength between the frequency bands from two different muscles, where the frequency bands nodes and links for each pair of muscles form a separate subnetwork (Figs. 3, 4, 5). Links strength is marked by line color and width – we use distinct link color-code to demonstrate network reorganization with fatigue. To represent how network organization changes with accumulation of fatigue during Exercise 1 (Fig. 3) and with residual fatigue across repeated exercise bouts (Exercise 1, 2, 3; Figs. 4 and 5) we use the following links strength classification: weak links (0.20 < C_i,j_ < 0.35; very thin grey lines), intermediate links (0.35 < C_i,j_ < 0.5; thin green lines), strong links (0.5 < C_i,j_ < 0.65; dark blue thick lines) and very strong links (C_i,j_ > 0.65; magenta very thick lines). To illustrate the contrast among different subnetworks, we use a different links strength classification. For same-type muscle subnetworks (Figs. 7 and 8): weak links (0.4 < C_i,j_ < 0.5; very thin grey lines), intermediate links (0.5 < C_i,j_ < 0.6; thin green lines), strong links (0.6 < C_i,j_ < 0.7; dark blue thick lines) and very strong links (C_i,j_ > 0.7; magenta very thick lines). For different-type muscle subnetworks (Fig. 9): weak links (0.25 < C_i,j_ < 0.3; very thin grey lines), intermediate links (0.3 < C_i,j_ < 0.35; thin green lines), strong links (0.35 < C_i,j_ < 0.4; dark blue thick lines) and very strong links (C_i,j_ > 0.4; magenta very thick lines). Links corresponding to cross-correlation values C_ij_ < 0.35 (Figs. 3, 4, 5), C_ij_ < 0.4 (Figs. 7, 8), C_ij_ < 0.25 (Fig. 9) are not shown in the network maps (see Methods 2.6.).

To visualize the hierarchical organization of intermuscular network interactions among muscle fibers (frequency band F_i_) from different muscles, the entire multiplex networks for the Beginning and End segments of Exercise 1 (Fig. 3a), as well as for Exercise 1, Exercise 2 and Exercise 3 (Fig. 4a) are presented separately as subnetwork maps for all pairs of muscles and distinct modules within the subnetworks. Each subnetwork map corresponds to a muscle pair for a given Exercise set and follows the same color-code as in the original network. We obtain two types of subnetworks: (i) same-type muscle subnetworks (ChestL-ChestR and ArmL-ArmR; Figs. 3b and 4b), and (ii) subnetworks of different muscle types (ChestL-ArmL, ChestL-ArmR, ChestR-ArmL and ChestR-ArmR; Fig. 5b). For all subnetworks, we show selected network modules for frequency bands F1, F3, F5, F8 and F10 that represent the activation of type-I, type IIa and type IIb muscle fibers.

To further dissect intermuscular interactions, we obtain stratification profiles of links strength for each subnetwork, and we track how the stratification profiles are modulated with accumulation of fatigue during Exercise 1 (Fig. 3) and with residual fatigue across repeated exercise bouts (Figs. 4 and 5). For each protocol segment/bout and all subnetwork representing different pairs of muscles, we calculate the average links strength for distinct modules within the subnetwork (corresponding group-averaged cross-correlation matrix in Fig. 2a). Considering that each subnetwork represents the interactions between EMG frequency bands for a given pair of muscles, we analyze the following network modules of cross-frequency coupling in each subnetwork: *Low-low* frequencies ([F1-F2]—[F1-F2]) bands interactions: average value of the coupling (matrix elements in Fig. 1d) between frequency bands [F1-F2] from one muscle with [F1-F2] from another muscle; *Low-intermediate* frequencies ([F1-F2]—[F3…F7]) bands interactions: average value of the coupling between frequency bands [F1-F2] from one muscle with [F3, F4, F5, F6, F7] from another muscle; *Low-high frequencies* ([F1-F2]—[F8…F10]) bands interactions: average value of the coupling between frequency bands [F1-F2] from one muscle with [F8, F9, F10] from another muscle; *Intermediate-intermediate* frequencies ([F3…F7]—[F3…F7]) bands interactions: average value of the coupling between frequency bands [F3, F4, F5, F6, F7] from one muscle with [F3, F4, F5, F6, F7] from another muscle; Intermediate-high frequencies ([F3…F7]—[F8…10]) bands interactions: average value of the coupling between frequency bands [F3, F4, F5, F6, F7] from one muscle with [F8, F9, F10] from another muscle; *High-high* frequencies ([F8…F10]—[F8…F10]) bands interactions: average value of the coupling between frequency bands [F8, F9, F10] from one muscle with [F8, F9, F10] from another muscle (Figs. 3c, 4c and 5b).

### Statistics and reproducibility

Statistical analyses were performed using SPSS (version 23; SPSS, Inc.). We find a non-gaussian distribution for the cross-frequency coupling values obtained for the individual subjects in our database (see boxplots for the links strength in different network modules presented in Fig. 6). Thus, to assess effects of accumulation of fatigue during Exercise 1 (Beginning vs End segment), and residual fatigue across Exercise 1, Exercise 2 and Exercise 3 on average links strength for the individual network modules we perform a non-parametric Wilcoxon matched-pairs test. To account for multiple tests correction in Figs. 4, 5, 8 and 9 (Exc 1 vs Exc 2, and Exc 1 vs Exc 3), we have applied a Wilcoxon matched-pairs test with Bonferroni correction (alpha level 0.025).

Our study aims to identify hierarchical organization in the intermuscular network of muscle fiber interactions where subnetworks are composed of distinct modules. Thus, we quantify average links strength for the individual network modules, as represented by the bar charts in Figs. 3d, 4d, 5d, 7, 8, 9, where we compare two conditions (Beginning vs End segment, and Exercise 1 vs Exercise 3) to test for the effects of accumulated and residual fatigue. Correspondingly, we focus only on pair wise comparison where the average links strength of a given module at the Beginning is compared with the links strength of the same module under fatigue for the End segment of Exercise 1.

To verify the reproducibility of the experimental findings, we compared the data: (i) within each participant and (ii) across participants. The findings are consistent within participants and across participants. To promote reproducibility among other researchers, we utilized Matlab_2022a’s analytic tools to obtain spectral power distribution and cross-correlation values (see Methods for a detailed explanation).

### Reporting summary

Further information on research design is available in the Nature Portfolio Reporting Summary linked to this article.

### Supplementary information

Description of Additional Supplementary Files

Supplementary Data

Reporting Summary

## Data Availability

The datasets generated during and/or analysed during the current study are available from the corresponding author on reasonable request by qualified researchers. The numerical source data for graphs and charts can be found in the Supplementary Data file

## Code availability

Matlab codes for data processing are available upon request from the corresponding author. Standard Matlab functions are used to perform the analyses (Fourier Transform, band-pass filtering in different frequency bands, and cross-correlation). These functions are available in the Matlab library.
