# Supplementary material for: Network of muscle fibers activation facilitates inter-muscular coordination, adapts to fatigue and reflects muscle function
Source: Commun Biol. 2023 Aug 30;6:891. doi: 10.1038/s42003-023-05204-3 (PMC10468525; doi:10.1038/s42003-023-05204-3)
Supplement: Supplementary file 1 — Description of Additional Supplementary Files [file 42003_2023_5204_MOESM1_ESM.pdf]

Description of Additional Supplementary Files

File name: Supplementary Data
Description: Numerical source data underlying the graphs and charts presented in the main figures
